# A fuzzy interval optimization approach for *p*-hub median problem under uncertain information

**DOI:** 10.1371/journal.pone.0297295

**Published:** 2024-03-15

**Authors:** Yu Wang, Tao Zhu, Kaibo Yuan, Xin Li

**Affiliations:** 1 School of Economics and Management, Civil Aviation Flight University of China, Guanghan, China; 2 College of Management Science, Chengdu University of Technology, Chengdu, China; Cyprus International University Faculty of Engineering: Uluslararasi Kibris Universitesi Muhendislik Fakultesi, TURKEY

## Abstract

Stochastic and robust optimization approaches often result in sub-optimal solutions for the uncertain *p*-hub median problem when continuous design parameters are discretized to form different environmental scenarios. To solve this problem, this paper proposes a triangular fuzzy number model for the Non-Strict Uncapacitated Multi-Allocation *p*-hub Median Problem. To enhance the quality and the speed of optimization, a novel optimization approach, combining the triangular fuzzy number evaluation index with the Genetic-Tabu Search algorithm, is proposed. During the iterations of the Genetic-Tabu Search algorithm for finding the optimal solution, the fitness of fuzzy hub schemes is calculated by considering the relative positional relationships of triangular fuzzy number membership functions. This approach directly addresses the triangular fuzzy number model and ensures the integrity of information in the *p*-hub problem as much as possible. It is verified by the classic Civil Aeronautics Board and several self-constructed data sets. The results indicate that, compared to the traditional Genetic Algorithm and Tabu Search algorithm, the Genetic-Tabu Search algorithm reduces average computation time by 49.05% and 40.93%, respectively. Compared to traditional random, robust, and real-number-based optimization approaches, the proposed optimization approach reduces the total cost in uncertain environments by 1.47%, 2.80%, and 8.85%, respectively.

## 1. Introduction

The *p*-hub median problem is one of the hub location problems that involves determining the locations of *p* hubs, the itineraries of all Origin-to-Destination (OD) pairs, and the link relations between points, such that the total system cost is minimized. Such kind of a problem can be further divided into Single Allocation *p*-Hub Median Problem (SApHMP) and Multiple Allocation *p*-Hub Median Problem (MApHMP) based on how hubs and non-hubs are connected [1]. Early interests in such kind of a problem were mainly focused on modeling skills. O’Kelly [2] proposed a nonlinear programming model with a quadratic objective function. Building upon the former research, Campbell [3] proposed a Non-Strict Uncapacitated Multi-Allocation *p*-Hub Median Problem (NSUMApHMP), which specifies that non-hubs can be interconnected and multiple paths can be selected for transportation. To efficiently address the classical *p*-hub center problem, Skorin-Kapov [4] developed new mixed linear formulations with a tight linear programming relaxation which effectively simplifies the model’s complexity. The aforementioned research developed several classical models of the *p*-hub median problem.

However, the assumption of deterministic design parameters in hub design models above is unrealistic and problematic due to inherent uncertainty, and will inevitably lead to sub-optimal selection of hub locations. To improve the anti-risk capability in uncertain environments, many scholars attempt to treat these design parameters (such as unit flow cost and demand, etc.) as random variables to generate environment scenarios. Stochastic optimization was one of the main effective approaches. It selected the hub location with the best expectation value for all scenarios. Typical representative findings are included as follows. Contreras et al. [5] solved the uncapacitated multiple allocation *p*-hub median problem (UMApHMP) by turning the random expectation model into a deterministic expectation model with known probability distributions. Zhai et al. [6] developed a two-stage model that serves the demand to solve the UMApHMP by minimizing the expected cost in each scenario. Ghaderi and Rahmaniani [7] modeled the uncapacitated single allocation p-hub median problem (USApHMP) with stochastic demand and travel time to obtain the optimal expectation value of the objective function in each scenario. Peiró et al. [8] developed a stochastic programming modeling framework for the r-allocation *p*-hub median problem with nonstop services. Based on these stochastic optimization models, heuristic algorithms are proposed to improve their ability to solve. Azizi et al. [9] proposed an exact algorithm and a Genetic Algorithm to solve the model that regards the total expected congestion cost as the objective function of USApHMP. Shang et al. [10] developed a memetic algorithm to solve MApHMP with stochastic flow and demand. Alvarez et al. [11] designed a meta-heuristic framework with Monte Carlo simulation to solve SApHMP, in which flows and demands are regarded as stochastic variables. These above researches provide feasible approaches to solve the uncertainty in the *p*-hub median problem.

Stochastic optimization methods typically necessitate specifying the exact probabilities for each environmental scenario, which is challenging in reality due to the inability to predefine all possible scenarios. Hence, scholars have introduced robust optimization methods, which optimize hub locations by searching for the best solutions under the worst-case scenarios, accommodating partial situations. Typical representative findings are included as follows. Ghaffarinasab [12] optimized the UMApHMP by minimizing the worst-case cost with the hybrid uncertainty set. Wang et al. [13] presented a novel two-stage distributed robust optimization model by minimizing the worst-case cost for the two-allocation *p*-hub median problem under the case that the demand has partially distributed information. Meraklč and Yaman [14] used the hose uncertainty set to minimize the worst-case cost within all possible demands for UMApHMP. These approaches obtain the optimal solution in the worst-case scenario by robust optimization models. To further solve the robust optimization model, Ghaffarinasab et al. [15] utilized the hybrid demand uncertainty sets to transform the robust USApHMP model into a linear MIP model, and the Tabu-based mate-heuristic solution algorithms were proposed to solve it. To construct a mean-robustness programming model to solve USApHMP, Ge [16] minimized the average of total cost and the weighted sum of risks in all scenarios. Ghaffarinasab [17] devised a precise solving algorithm based on Benders decomposition to address the UMApHMP. These stochastic optimization researches above provide feasible approaches to the *p-*hub median problem under uncertain cases with unknown scenarios.

The above two aforementioned approaches partially address the uncertainty in the *p*-hub median problem. However, due to their limited utilization of continuous parameter information and lack of specification of distribution probabilities, the generated results are likely to remain sub-optimal [18]. Many scholars attempted to use interval-valued data and fuzzy mathematical methods to solve the hub location problem. Therefore, the interval-valued numbers with upper and lower limits are introduced into the study. Ghaffari-Nasab et al. [19] represented the uncertain parameters by the midpoint of the two interval-valued numbers to solve the capacitated single and multiple allocation hub location problems. Habibzadeh Boukani et al. [20] transformed a kind of two interval-valued parameter model into an exact real number type-based one using the expectations of the interval cost and capacity. Zetina et al. [21] developed an exact real number type-based model to represent the uncertain parameters with the interval width values of the two interval-valued parameters. Talbi and Todosijevic [22] quantified the solution uncertainty by the widths of two interval parameters. Similarly, some studies generated the interval values of parameters such as the travel time to overcome the shortcomings of the *p*-hub model. Amin-Naseri et al. [23] established a robust bi-objective uncapacitated single allocation *p*-hub median problem by setting the minimization of travel time uncertainty as the objective function. De sá [24] introduced uncertain budget factors and set demand and travel time as interval numbers to overcome the defects of exact models. These above researches on interval numbers successfully leverage the continuous information of parameters in the *p*-hub median problem, rather than relying solely on a finite set of discrete scenarios.

To raise the accuracy of the decision results, decision-makers should provide not only the upper and lower bounds but also the most probable values of related parameters while selecting the location of hubs. Triangular fuzzy numbers can more realistically represent the parameters of hub location problems. Therefore, many Scholars construct a triangular fuzzy number and convert it into a precise model for a solution. Maharjan [25] transformed the temporary logistics hub location-allocation model with triangular fuzzy numbers into an exact real number type-based one based on a fuzzy chance constraint of the credibility distribution theory. Kayvanfar [26] proposed a method to solve the multi-objective and multi-echelon supply-distribution model based on the fuzzy triangular reference set weight. TootoonI et al. [27] developed triangular fuzzy type I and II programming approaches with interval models to solve the single allocation ordered median hub location problem. These above researches use triangular fuzzy numbers to solve the of hub-location allocation problem. Furthermore, in terms of path allocation, Tirkolaee et al. [28] described the demand as a triangular fuzzy number and established a fuzzy chance-constrained programming model to solve the fuzzy multi-trip location-routing problem. Sun [29] employed a defuzzification, linearization, and weighted sum method to present a crisp linear model to solve the routing problem in the road-rail intermodal transportation network. Compared with interval numbers, these methods with triangular fuzzy numbers fully utilize the information of the parameters in the p-hub median problem.

Through a comprehensive review of studies on the *p*-hub median problem under uncertain conditions, the following conclusions can be drawn: (1) Current research often transforms interval models into various precise real-number models for solution purposes. (2) Existing algorithms have not directly addressed mathematical programming models with triangular fuzzy number parameters. These research frameworks only leverage partial characteristics of triangular fuzzy number parameters, rather than considering their complete information, which may compromise solution accuracy. To further enhance the quality and precision of decision solutions, this study proposes a novel optimization approach building upon the models and algorithms employed by predecessors in addressing the *p*-hub median problem. This approach integrates fuzzy mathematics theory with heuristic algorithms, incorporating the membership function evaluation index of triangular fuzzy numbers into heuristic algorithms. Consequently, it establishes an algorithm capable of fully utilizing information from uncertain parameters and directly solving the *p*-hub median problem with triangular fuzzy numbers. In comparison to prior work, the contributions of the present study can be summarized as follows.

(1) A fuzzy NSUMApHMP model based on the triangular fuzzy number is proposed, capable of determining the optimal hub network under uncertain conditions and revealing paths with the lowest cost at different scales of the *p*-hub median problem.

(2) A customized heuristic algorithm is devised with a triangular fuzzy number evaluation index as the fitness operator, allowing for the direct solution of the triangular fuzzy number *p*-hub median problem. The evaluation index for triangular fuzzy numbers is based on the relative positional relationships derived from membership function deductions, employed to assess the cost-effectiveness of hub location schemes. This customized heuristic algorithm is an improved Genetic-Tabu Search algorithm utilizing an enhanced Floyd-Warshall algorithm to explore the shortest paths for all OD pairs in the hub network.

The remaining sections of this paper are organized as follows. Section 2 introduces an NSUMApHMP model based on triangular fuzzy numbers. A fuzzy evaluation index is constructed as a fitness operator to assess the merits of various fuzzy hub schemes. Section 3 devises a Genetic-Tabu Search algorithm, considering the proposed evaluation index as a fitness operator. In Section 4, the effectiveness of the proposed algorithm and optimization approach is validated based on the classical Civil Aviation Bureau (CAB) dataset and several self-generated datasets. The sensitivity analysis verifies the impact of triangular fuzzy number type characteristics on the hub scheme. Finally, Section 5 concludes the entire paper.

## 2. Mathematical formulation

This research aims to generate high-quality decision-making solutions to the *p-*hub media problem by fully utilizing the information in triangular fuzzy number parameters. Therefore, a unique optimization approach based on a domain search heuristic algorithm is proposed. During the domain search process, the triangular fuzzy number evaluation index is used as a fitness operator to sort the cost of hub solutions. This approach can solve the interval model of the *p*-hub medium problem directly, avoiding the information loss caused by converting the interval model into an accurate real number model. This section introduces the research problem and mathematical model first, and then the establishment process of the evaluation index for the hub scheme of triangular fuzzy number type as a fitness operator in the algorithm.

### 2.1. Model description and assumptions

The advantage of Non-Strict Uncapacitated Multi Allocation *p*-hub Median Problem (NSUMApHMP) in *p*-hub median problem is that it allows for the allocation of multiple hubs, which results in better adaption to complex transportation networks and demand distribution in reality [30]. Based on this model, this study proposed a novel fuzzy NSUMApHMP model considering passenger flow and cost as uncertain parameters. In this model, triangular fuzzy numbers are used to represent uncertain passenger flow and cost, in order to better fit the real air transportation situation. By incorporating triangular fuzzy numbers into the model, the utilization of continuous parameters in the optimization-solving process is ensured, thereby avoiding the information loss caused by parameter discretization. The aim of the fuzzy NSUMApHMP model is to determine the locations of *p* hub cities, the allocation of non-hub cities to hub cities, and the transfer flow ratio of each OD pair (*i*,*j*) via hubs *k* and *m*, with the objective of minimizing the total system cost. Following classic assumptions listed in Campbell [3] and O’Kelly [2], the assumptions of our paper can be depicted as follows.

(1) Assume that all hubs are fully connected to each other. Each non-hub node can be connected to any multiple hub nodes, and the non-hub nodes can also be directly connected to each other.

(2) Suppose that any one OD passenger flow can be transferred between two hub nodes at most.

(3) The hub’s capacity and the hub network’s links are infinite.

(4) The number of required hubs to locate is fixed in advance.

(5) Assume that there are economies of scale between hub nodes such that the discount factor can be applied to the unit flow cost of routing between a pair of hubs.

(6) The design parameters including the unit flow cost and demand are *α* (0≤*α*≤1) considered to be the triangular fuzzy number types.

### 2.2. Notations

(1) *D*: the total number of nodes in a network.

(2) *p*: the number of selected hubs.

(3) ${\tilde{W}}_{ij}$: the passenger demand volume between node *i* and *j*, in the form of triangular fuzzy number type, generally has ${\tilde{W}}_{\mathrm{ij}}\neq {\tilde{W}}_{ji}$, but for simplicity, it assumes ${\tilde{W}}_{\mathrm{ij}}={\tilde{W}}_{ji}$.

(4) ${\tilde{c}}_{ij}$: the unit flow cost from node *i* directly to node *j*, in the form of triangular fuzzy number type.

(5) ${\tilde{C}}_{ijkm}$: the unit flow cost of transporting a passenger from node *i* to node *j* via hub k and m, where ${\tilde{C}}_{ijkm}={\tilde{c}}_{ik}+\alpha {\tilde{c}}_{km}+{\tilde{c}}_{mj}$.

(6) *X*_*ijkm*_: the proportion of an OD passenger demand volume transported via path *i*→*k*→*m*→*j* (where *k*, *m* are hubs) to the total OD passenger demand volume.

(7) *Z*_*ij*_: the proportion of an OD passenger demand volume directly transported to the total OD passenger demand volume.

(8) *Y*_*k*_: 1 if node *k* is selected as the hub, 0 otherwise.

### 2.3. Mathematical model

The triangular fuzzy number type-based NSUMA*p*HMP model based on Campbell’s 4-index model [3] can be written as follows.

$$Min\;Z=\sum_{i\in N}\sum_{j\in N}\sum_{k\in N}\sum_{m\in N}{\tilde{W}}_{ij}X_{ijkm}{\tilde{C}}_{ijkm}+\sum_{i\in N}\sum_{j\in N}{\tilde{W}}_{ij}Z_{ij}{\tilde{C}}_{ij}$$ (1)

*s*.*t*.

$$\sum_{k\in N}Y_{k}=p$$ (2)

$$Z_{ij}+\sum_{k\in N}\sum_{m\in N}X_{ijkm}=1,\forall i,j\in D$$ (3)

$$X_{ijkm}\leq Y_{k},\;\forall i,j,k,m\in D$$ (4)

$$X_{ijkm}\leq Y_{m},\;\forall i,j,k,m\in D$$ (5)

$$Y_{k}\in \{0,1\},\forall k\in D$$ (6)

$$0\leq X_{ijkm}\leq 1,\;\forall i,j,k,m\in D$$ (7)

$$0\leq Z_{ij}\leq 1,\;\forall i,j\in D$$ (8)

The objective function is to minimize the sum of the transportation cost of the hub scheme of the triangular fuzzy number type. Constraint (2) ensures that the total number of hubs selected equals p. Constraints (3) ensure that each OD passenger demand volume can be totally transported. Constraints (4)-(5) ensure that each non-hub node can be connected to multiple hub nodes. Constraints (6)-(8) indicate the type and the range of values of decision variables.

The objective function of this model contains parameters of the triangular fuzzy number type, and it is apparent that the proposed model preserves the full information of these uncertain parameters. However, this makes the solution challenging as the objective function of the proposed model is the total transportation cost of triangular fuzzy number type, which cannot be directly solved using linear programming methods. To assess the superiority of the fuzzy hub scheme and obtain the global optimal solution, an evaluation index and a customized algorithm are required.

### 2.4. Triangular fuzzy number evaluation index based on membership function

To design a heuristic algorithm based on triangular fuzzy numbers, it is necessary to compare the fitness of each hub scheme of the triangular fuzzy number type during the algorithm iteration process. The following subsection provides an evaluation index that can be used to compare the cost of hub schemes in any scenario and rank multiple hub schemes. By comparing the relative positions of membership functions for transport cost based on triangular fuzzy numbers, a comparison of hub schemes is conducted.

#### 2.4.1. Basic definition

**Definition** 1 [31] Let $\tilde{A}=(A^{L},A^{M},A^{N})$ be said as a triangular fuzzy number where $0\leq A^{L}\leq A^{M}\leq A^{N}$. The lower bound of $\tilde{A}$ is defined as *A*^*L*^, the upper bound of $\tilde{A}$ is represented as *A*^*N*^ and the most probable value of $\tilde{A}$ is expressed as *A*^*M*^. Characteristic membership function of $\tilde{A}$ can be expressed as Eq (9).

$$\mu_{\tilde{A}}(x)=\{\begin{array}{l} \frac{x-A^{L}}{A^{M}-A^{L}}\;A^{L}\leq x\leq A^{M}\\ \frac{x-A^{N}}{A^{M}-A^{N}}\;A^{M}\leq x\leq A^{N}\\ \;0\;otherwise \end{array}$$ (9)

Note that when $A^{L}=A^{M}=A^{N}$, the triangular fuzzy number $\tilde{A}$ is indicated as an ordinary positive real number. Several definitions related to operators of triangular fuzzy numbers are shown as follows.

(1) Addition: $\tilde{A}+\tilde{B}=(A^{L},A^{M},A^{N})+(B^{L},B^{M},B^{N})=(A^{L}+B^{L},A^{M}+B^{M},A^{N}+B^{N})$.

(2) Multiplication: $\tilde{A}\tilde{B}=(A^{L}B^{L},A^{M}B^{M},A^{N}B^{N})$, $\lambda \tilde{A}=(\lambda A^{L},\lambda A^{M},\lambda A^{N})$ (*λ*>0).

**Definition 2** [32] Let *X* be a domain, and the set of all fuzzy sets on *X* is defined as *F*(*X*). If $\tilde{A}\in F(x)$ and ∃α∈[0,1], the definition of ${\tilde{A}}_{\alpha }$ can be written as Eq (10).

$${\tilde{A}}_{\alpha }=\{x\in X|\mu_{\tilde{A}}(x)\geq \alpha \}$$ (10)

It calls ${\tilde{A}}_{\alpha }$ as the *α*-cut of the fuzzy set $\tilde{A}$. Similarly, for ∀*α*∈[0,1], ${\tilde{A}}_{\alpha }=[A_{L}(\lambda ),A_{R}(\lambda )]$.

#### 2.4.2. Comparison of hub schemes of triangular fuzzy number type

**Fig 1:**
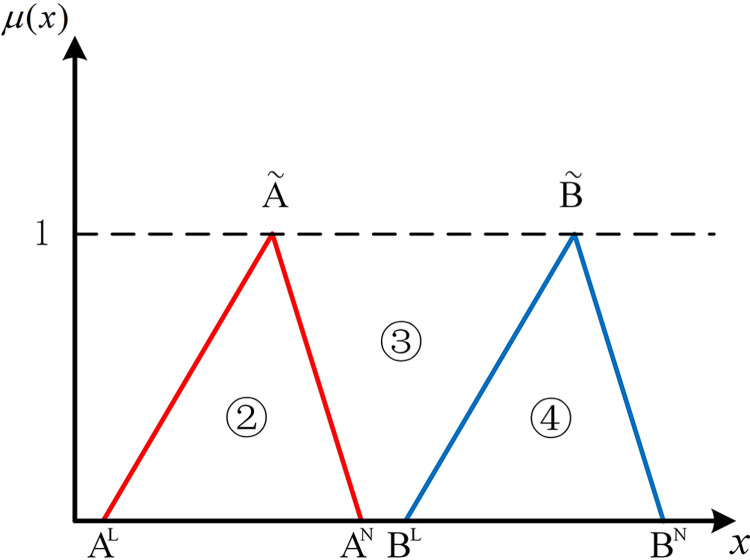
Scenario 1.

**Fig 2:**
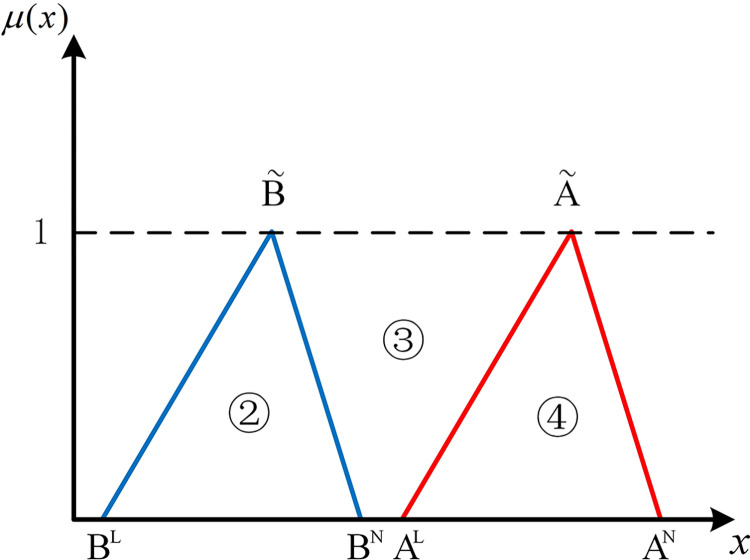
Scenario 2.

The positions of numbers besides two hub schemes of the triangular fuzzy number type ($\tilde{A}$ and $\tilde{B}$) can express the position relationship between them in the coordinate system. As shown in Fig 1, if there are no overlapping parts of two hub schemes of the triangular fuzzy number type, it’s easy to be denoted by Scenario 1 that $A^{N}<B^{L}$, all parts of $\tilde{B}$ are to the right of $\tilde{A}$. At this point, $\tilde{B}$ is strictly superior to $\tilde{A}$. As shown in Fig 2, in Scenario 2, if *B*^*N*^<*A*^*L*^, all parts of $\tilde{B}$ are to the left of $\tilde{A}$. Similarly, $\tilde{A}$ is strictly superior to $\tilde{B}$.

If two hub schemes of the triangular fuzzy number type overlap, all eight possible scenarios can be depicted from Figs 3–10. These eight specific scenarios can be expressed using the following formulas.

Scenario 3: $A^{L}\leq B^{L},A^{M}\leq B^{M},A^{N}\leq B^{N}$. Scenario 4: $A^{L}\leq B^{L},A^{M}\geq B^{M},A^{N}\leq B^{N}$.

Scenario 5: $A^{L}\leq B^{L},A^{M}\leq B^{M},A^{N}\geq B^{N}$. Scenario 6: $A^{L}\leq B^{L},A^{M}\geq B^{M},A^{N}\geq B^{N}$.

Scenario 7: $A^{L}\geq B^{L},A^{M}\leq B^{M},A^{N}\leq B^{N}$. Scenario 8: $A^{L}\geq B^{L},A^{M}\geq B^{M},A^{N}\leq B^{N}$.

Scenario 9: $A^{L}\geq B^{L},A^{M}\leq B^{M},A^{N}\geq B^{N}$. Scenario 10: $A^{L}\geq B^{L},A^{M}\geq B^{M},A^{N}\geq B^{N}$.

As shown above, all overlapping relationships between two hub schemes of the triangular fuzzy number type can be divided into at most six regions, namely ①, ②, ③, ④, ⑤, and ⑥.

**Fig 3:**
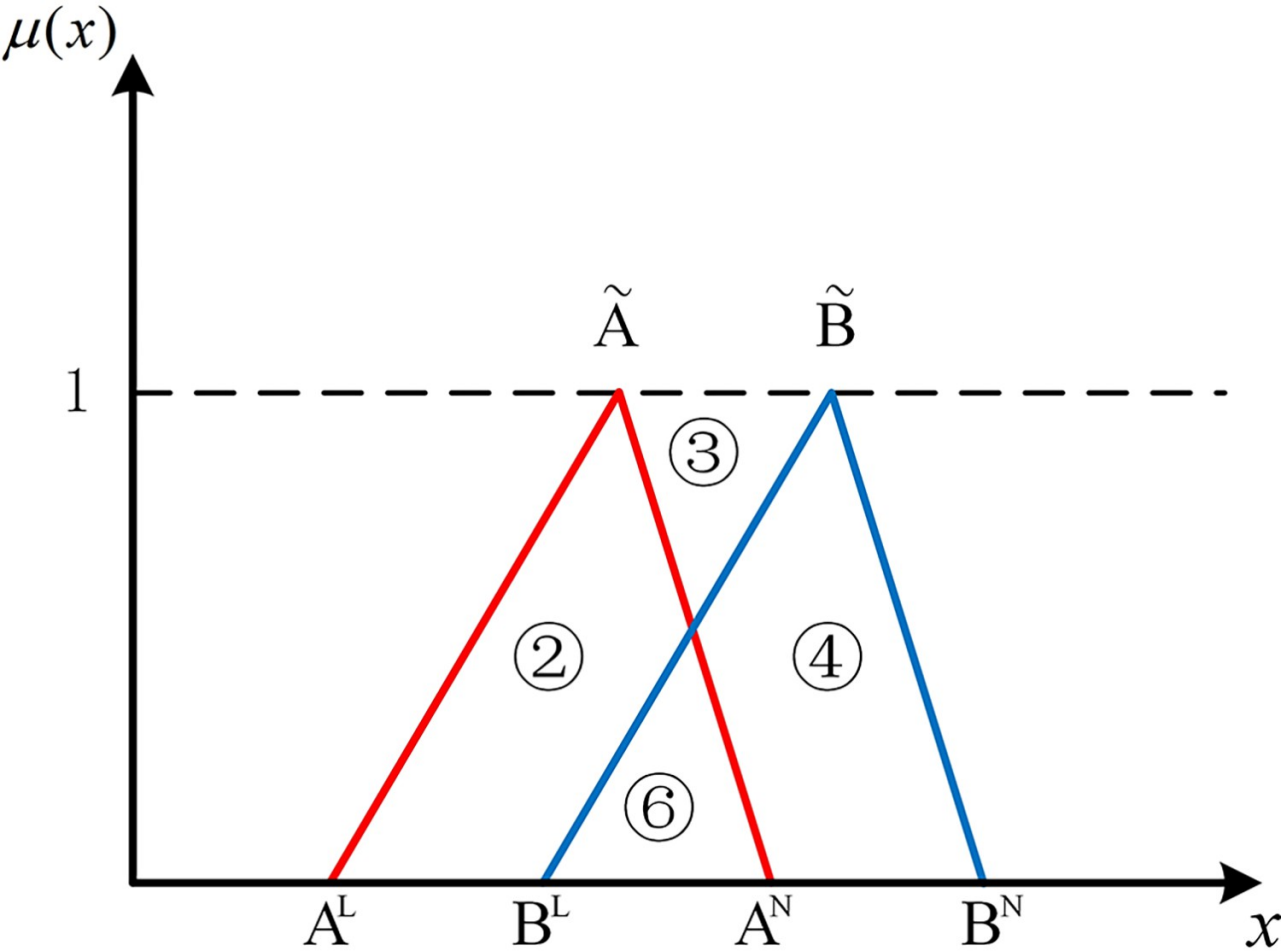
Scenario 3.

**Fig 4:**
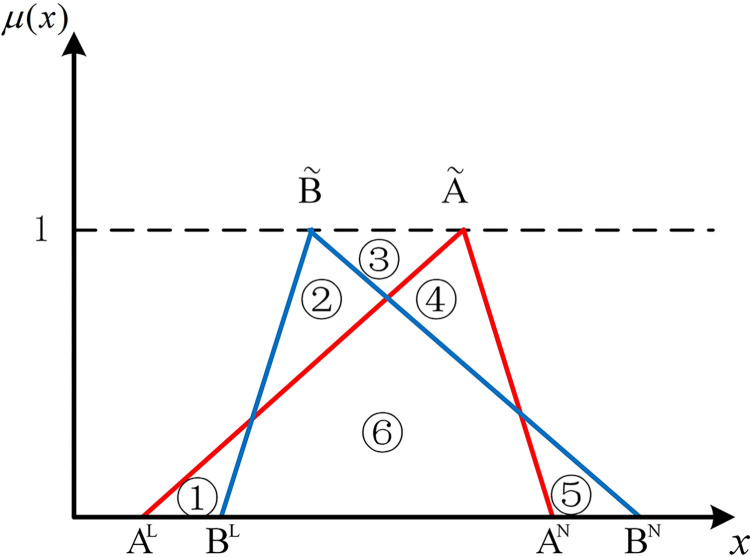
Scenario 4.

**Fig 5:**
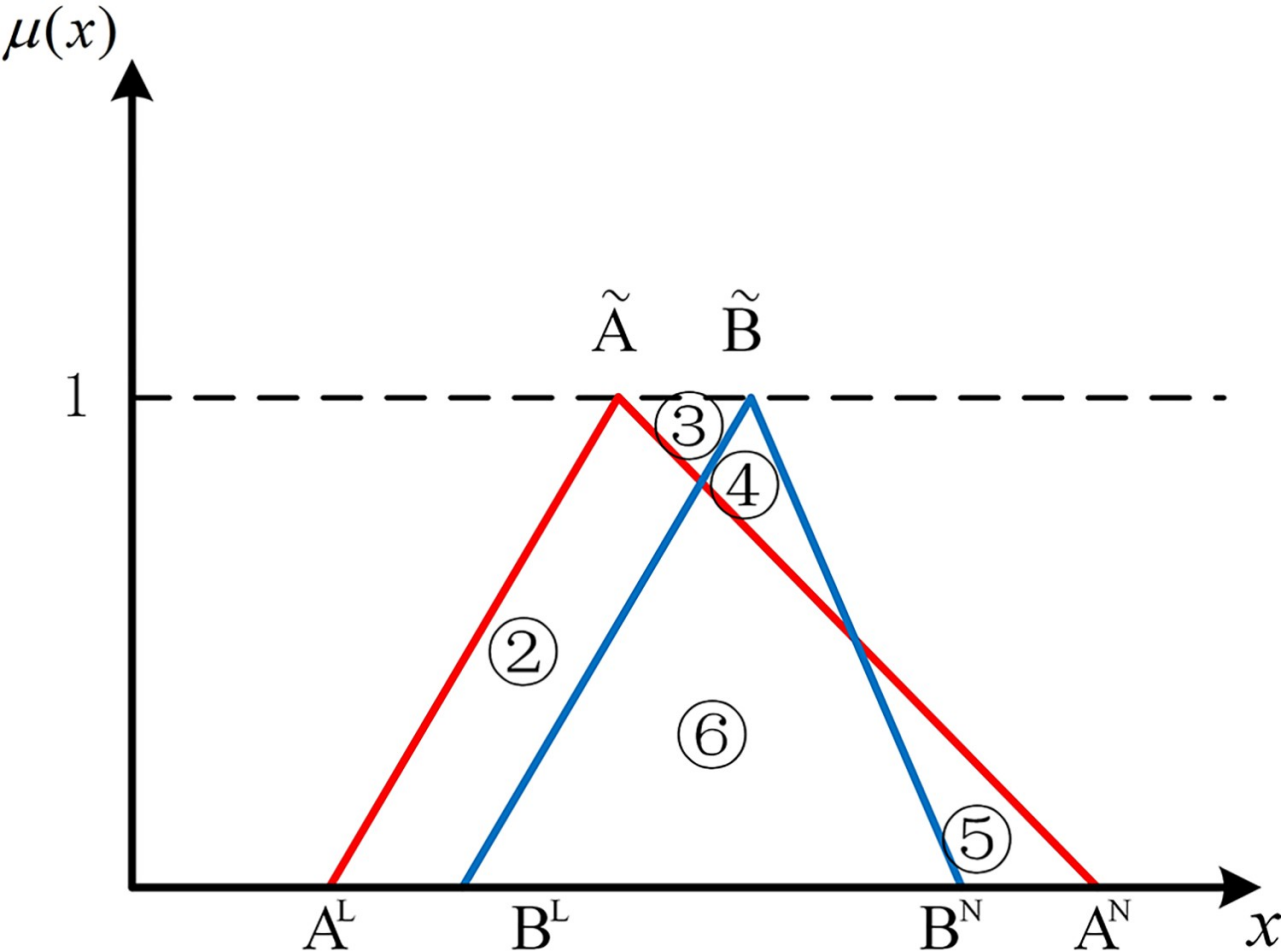
Scenario 5.

**Fig 6:**
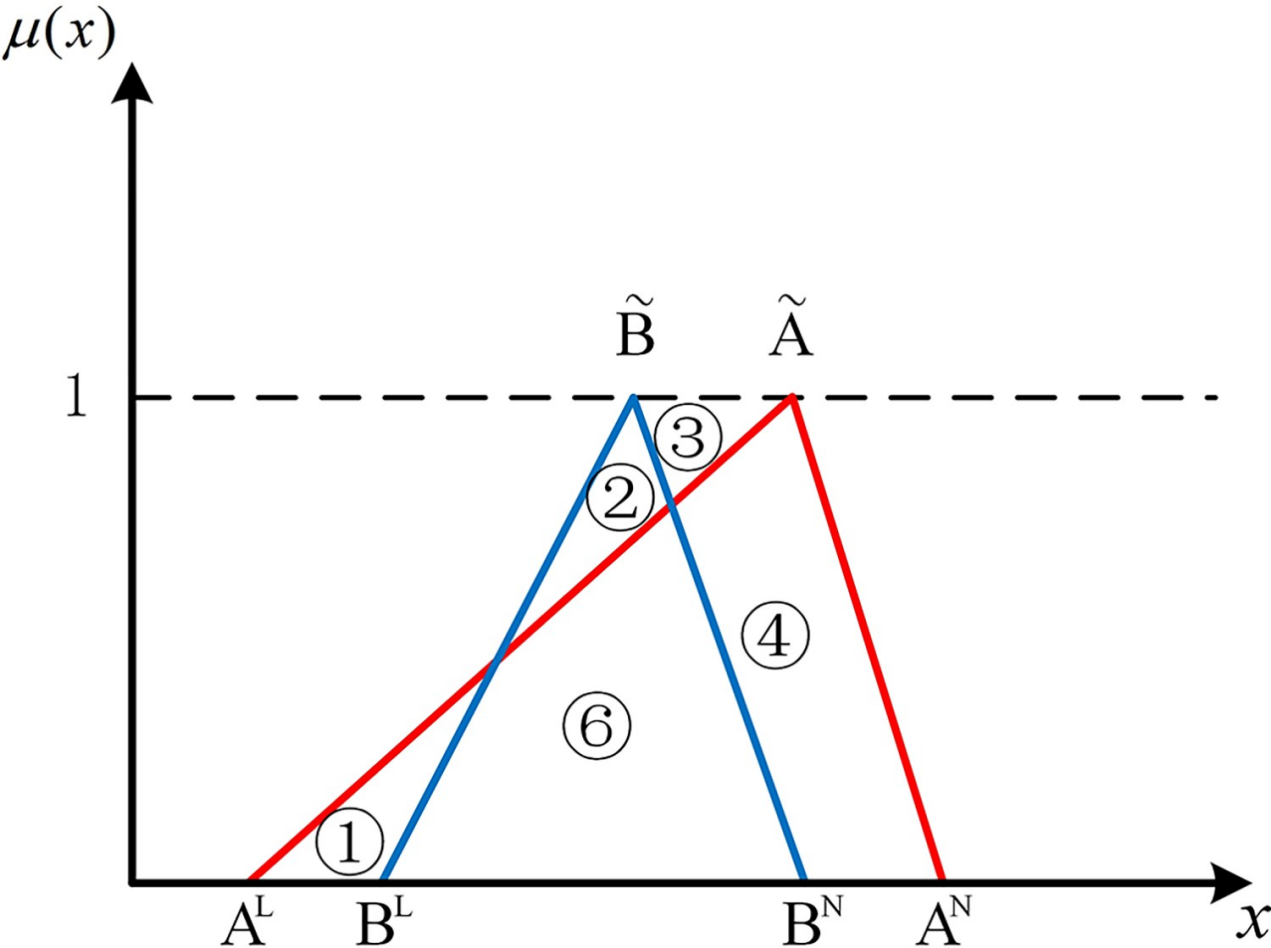
Scenario 6.

**Fig 7:**
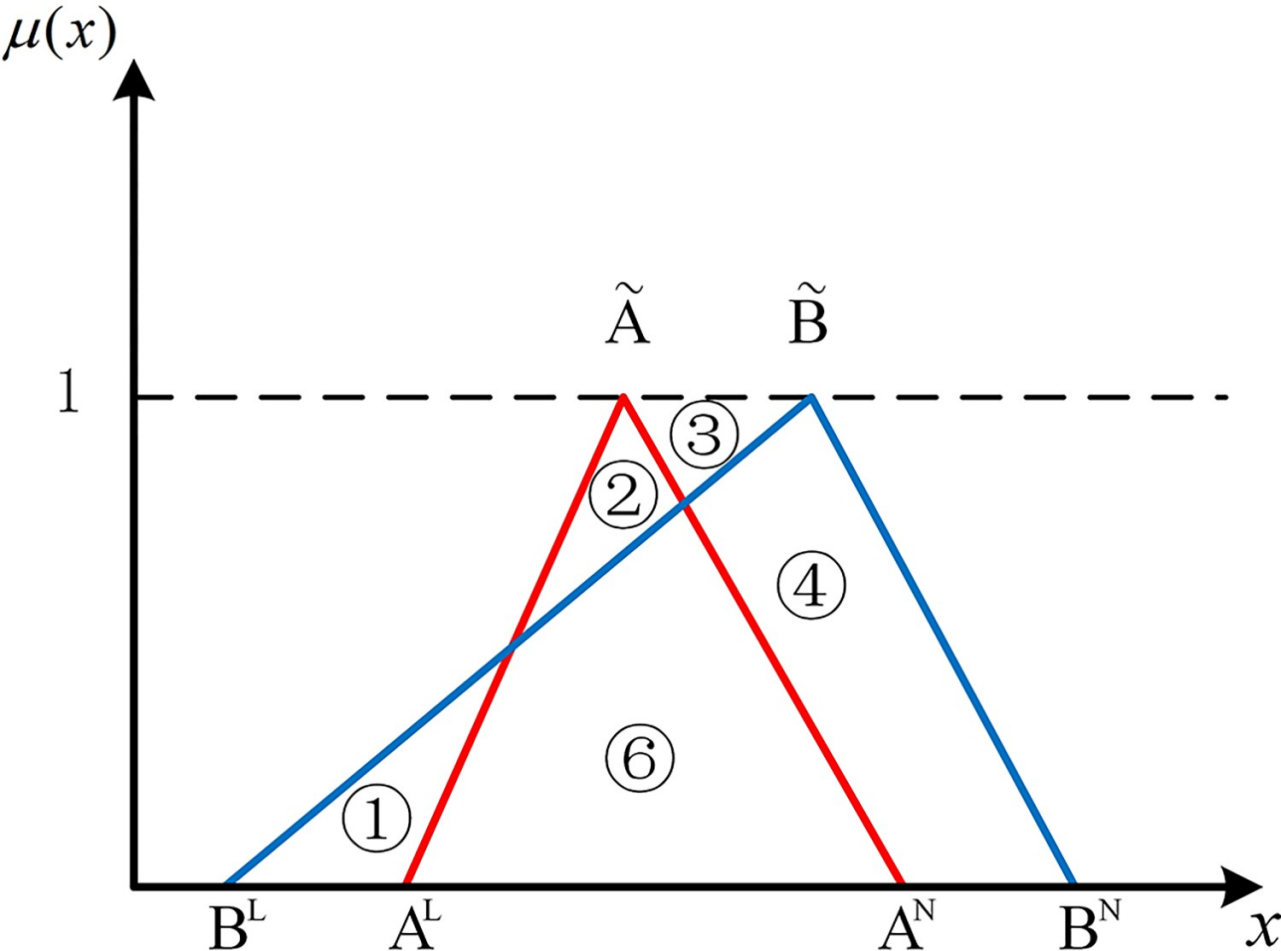
Scenario 7.

**Fig 8:**
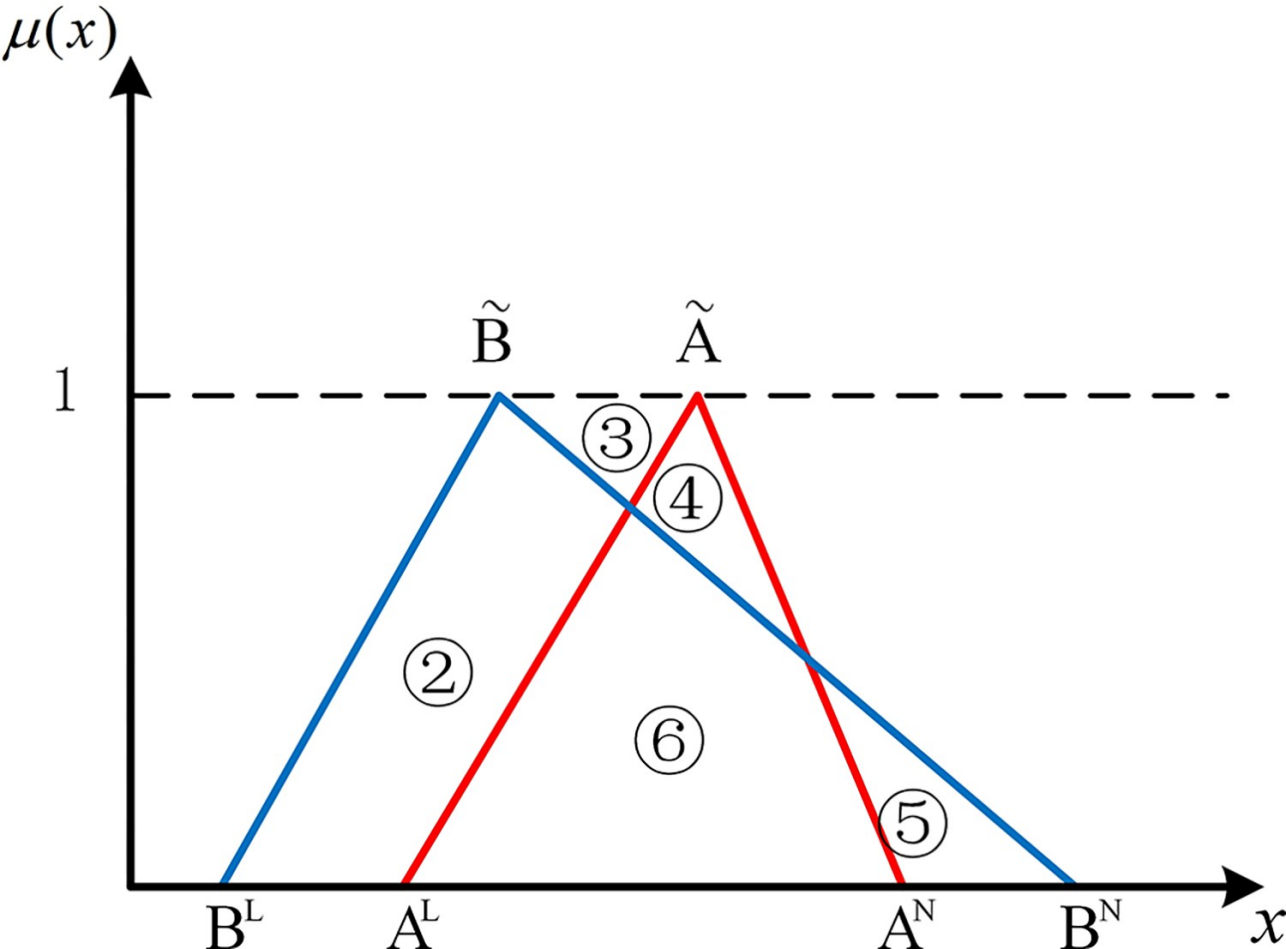
Scenario 8.

**Fig 9:**
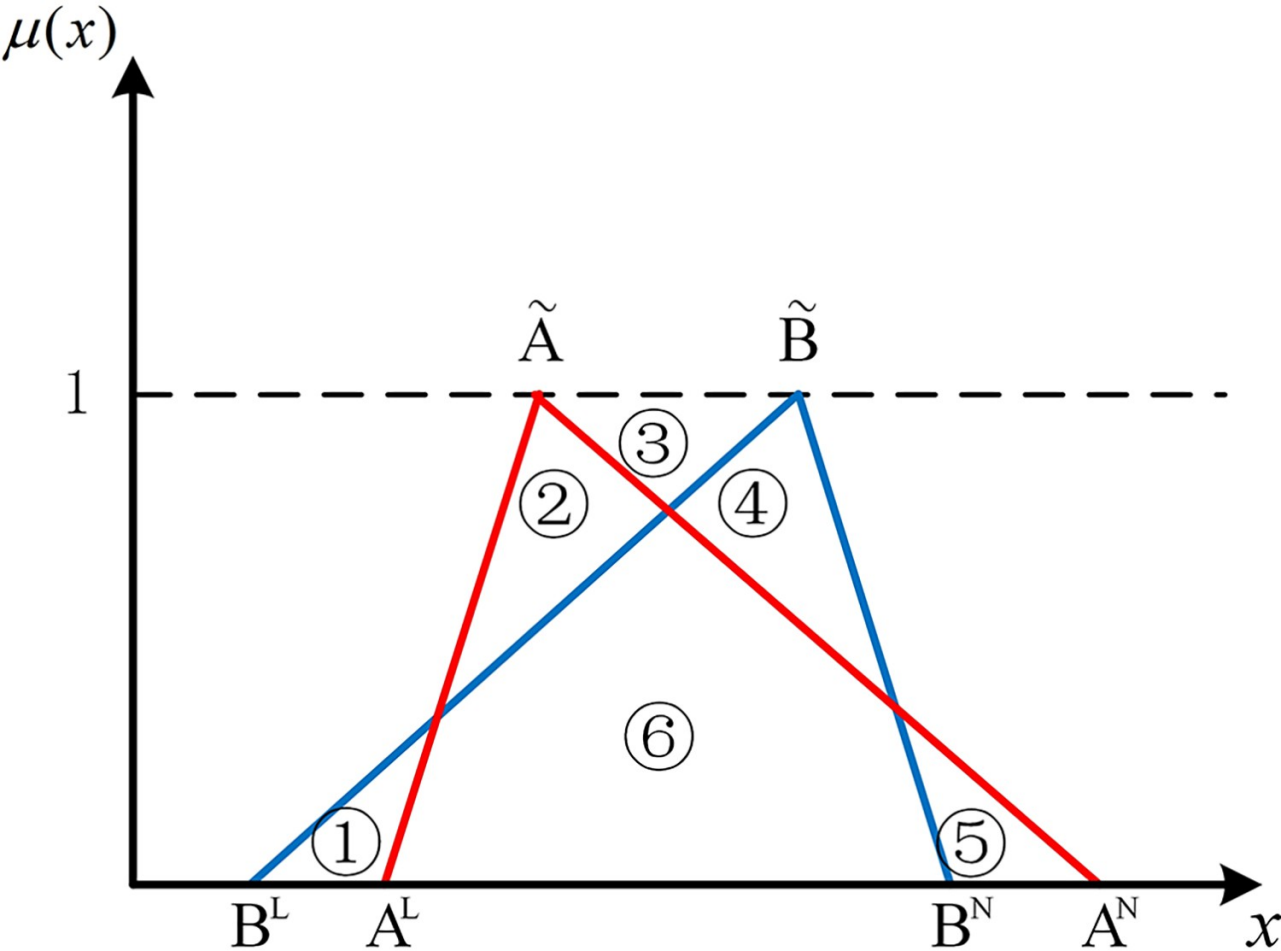
Scenario 9.

**Fig 10:**
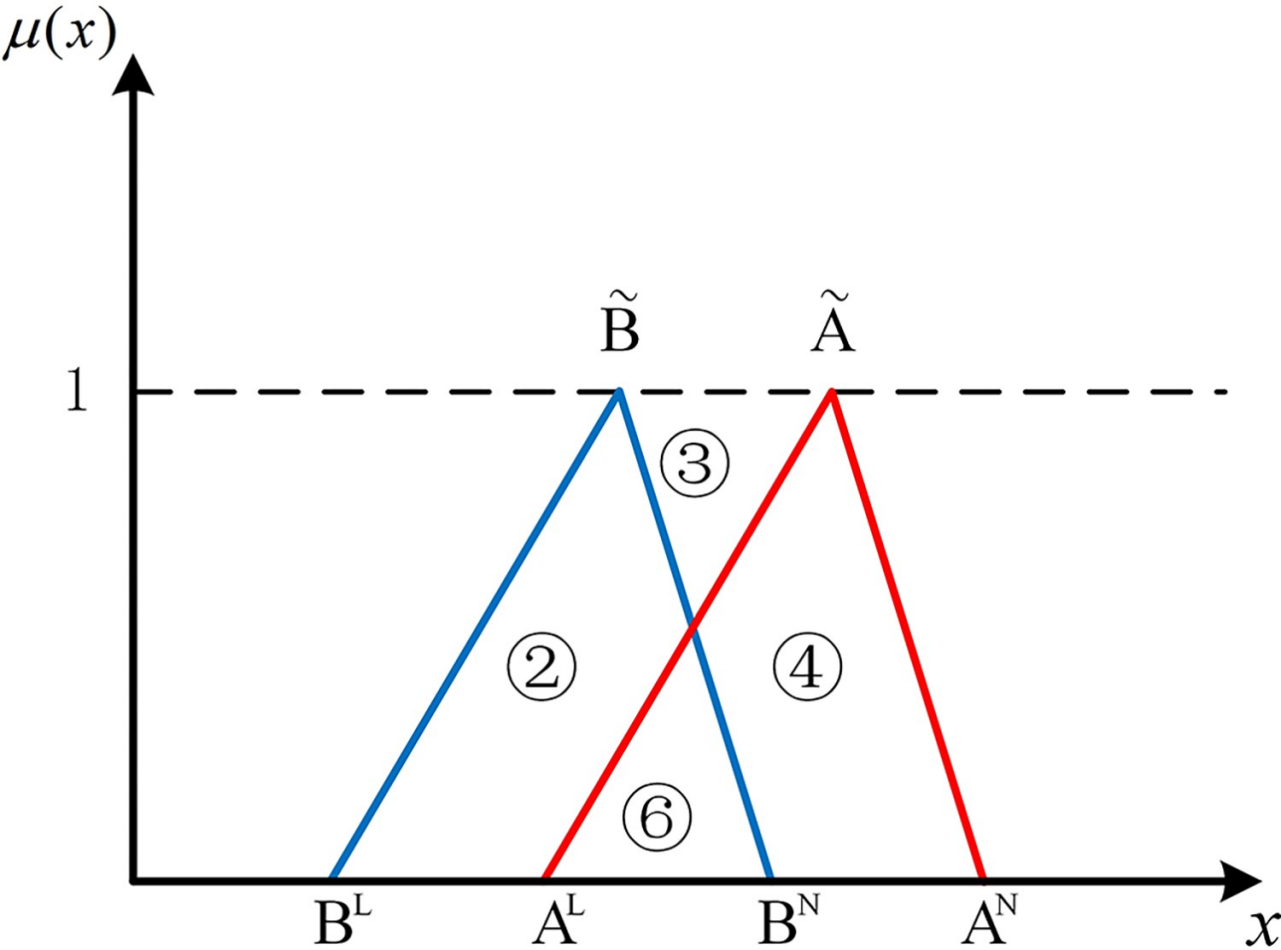
Scenario 10.

#### 2.4.3. Construction of the evaluation index based on position relationship of membership function

Based on the overlapping area of membership function, the fuzzy preference relations $N(\tilde{A},\tilde{B})$ can be expressed as

$$N(\tilde{A},\tilde{B})=\frac{S_{2}+S_{4}+S_{5}}{S_{1}+S_{2}+S_{3}+S_{4}+2S_{5}}$$ (11)

Geometrically, *S*_1_ shows the area of the left part of $\tilde{B}$ superior to the left part of $\tilde{A}$. *S*_2_ shows the area of the left part of $\tilde{A}$ superior to the left part of $\tilde{B}$. *S*_3_ shows the area of the right part of $\tilde{B}$ superior to the right part of $\tilde{A}$. *S*_4_ shows the area of the right part of $\tilde{A}$ superior to the right part of $\tilde{B}$. *S*_5_ shows the area of the overlapping part of $\tilde{A}$ and $\tilde{B}$.

For direct calculation, Eq (11) can be transformed into several mathematical expressions containing *A*^*L*^, *A*^*M*^, and *A*^*N*^ for $N(\tilde{A},\tilde{B})$ through *R*_*i*_ ({*R*_*i*_|*i*∈1,2,…6} corresponds to ①, ②, ③, ④, ⑤, and ⑥ in Figs 1–10, respectively), as shown in Table 1.

**Table 1:** Formulas and expressions for $N(\tilde{A},\tilde{B})$ from Scenarios 3 to 10.

Scenario	$N(\tilde{A},\tilde{B})$	The specific expression of $N(\tilde{A},\tilde{B})$
3	$\frac{R_{6}}{R_{2}+2R_{3}+R_{4}+2R_{6}}$	$\frac{{\left(A^{N}-B^{L}\right)}^{2}}{(A^{N}-B^{L}+B^{N}-A^{L})(A^{N}-B^{L}-A^{M}+B^{M})+2{\left(B^{M}-A^{M}\right)}^{2}}$
4	$\frac{R_{2}+2R_{3}+R_{4}+R_{6}}{R_{1}+R_{2}+2R_{3}+R_{4}+R_{5}+2R_{6}}$	$1-\frac{{\left(B^{N}-A^{L}\right)}^{2}}{{\left(B^{N}-A^{L}\right)}^{2}+(B^{N}-A^{L}-B^{M}+A^{M})(A^{N}-B^{L}-B^{M}+A^{M})+{\left(B^{M}-A^{M}\right)}^{2}}$
5	$\frac{R_{5}+R_{6}}{R_{2}+2R_{3}+R_{4}+R_{5}+2R_{6}}$	$\frac{{\left(A^{N}-B^{L}\right)}^{2}}{(A^{N}-B^{L}+B^{N}-A^{L})(A^{N}-B^{L}-A^{M}+B^{M})+2{\left(B^{M}-A^{M}\right)}^{2}}$
6	$\frac{R_{2}+2R_{3}+R_{4}+R_{6}}{R_{1}+R_{2}+2R_{3}+R_{4}+2R_{6}}$	$1-\frac{{\left(B^{N}-A^{L}\right)}^{2}}{{\left(B^{N}-A^{L}\right)}^{2}+(B^{N}-A^{L}-B^{M}+A^{M})(A^{N}-B^{L}-B^{M}+A^{M})+{\left(B^{M}-A^{M}\right)}^{2}}$
7	$\frac{R_{1}+R_{6}}{R_{1}+R_{2}+2R_{3}+R_{4}+2R_{6}}$	$\frac{{\left(A^{N}-B^{L}\right)}^{2}}{(A^{N}-B^{L}+B^{N}-A^{L})(A^{N}-B^{L}-A^{M}+B^{M})+2{\left(B^{M}-A^{M}\right)}^{2}}$
8	$\frac{R_{2}+2R_{3}+R_{4}+R_{6}}{R_{2}+2R_{3}+R_{4}+R_{5}+2R_{6}}$	$1-\frac{{\left(B^{N}-A^{L}\right)}^{2}}{{\left(B^{N}-A^{L}\right)}^{2}+(B^{N}-A^{L}-B^{M}+A^{M})(A^{N}-B^{L}-B^{M}+A^{M})+{\left(B^{M}-A^{M}\right)}^{2}}$
9	$\frac{R_{1}+R_{5}+R_{6}}{R_{1}+R_{2}+2R_{3}+R_{4}+R_{5}+2R_{6}}$	$\frac{{\left(A^{N}-B^{L}\right)}^{2}}{(A^{N}-B^{L}+B^{N}-A^{L})(A^{N}-B^{L}-A^{M}+B^{M})+2{\left(B^{M}-A^{M}\right)}^{2}}$
10	$\frac{R_{2}+2R_{3}+R_{4}+R_{6}}{R_{2}+2R_{3}+R_{4}+2R_{6}}$	$1-\frac{{\left(B^{N}-A^{L}\right)}^{2}}{{\left(B^{N}-A^{L}\right)}^{2}+(B^{N}-A^{L}-B^{M}+A^{M})(A^{N}-B^{L}-B^{M}+A^{M})+{\left(B^{M}-A^{M}\right)}^{2}}$

It can be further summarized as

$$\begin{array}{c} N_{3}(\tilde{A},\tilde{B})=N_{5}(\tilde{A},\tilde{B})=N_{7}(\tilde{A},\tilde{B})=N_{9}(\tilde{A},\tilde{B})\\ =\frac{{\left(A^{N}-B^{L}\right)}^{2}}{(A^{N}-B^{L}+B^{N}-A^{L})(A^{N}-B^{L}-A^{M}+B^{M})+2{\left(B^{M}-A^{M}\right)}^{2}}, \end{array}$$ (12)

and

$$\begin{array}{c} N_{4}(\tilde{A},\tilde{B})=N_{6}(\tilde{A},\tilde{B})=N_{8}(\tilde{A},\tilde{B})=N_{10}(\tilde{A},\tilde{B})\\ =1-\frac{{\left(B^{N}-A^{L}\right)}^{2}}{{\left(B^{N}-A^{L}\right)}^{2}+(B^{N}-A^{L}-B^{M}+A^{M})(A^{N}-B^{L}-B^{M}+A^{M})+{\left(B^{M}-A^{M}\right)}^{2}}. \end{array}$$ (13)

Note that there’s no area overlapping in Scenario 1 and Scenario 2 as shown in Figs 1 and 2, respectively. The corresponding values of $N(\tilde{A},\tilde{B})$ are obtained by Eq (11) equaling 0 and 1 respectively. We find the superiority of $\tilde{A}$ and $\tilde{B}$ in Scenario 1 is only related to area ③ as well as in Scenario 2. Therefore, two new formulas can be added to represent Scenario 1 and Scenario 2 (as shown in Table 2).

**Table 2:** Formulas and expressions for $N(\tilde{A},\tilde{B})$ from Scenarios 1 to 2.

Scenario	$N(\tilde{A},\tilde{B})$	The specific expression of $N(\tilde{A},\tilde{B})$
1	$\frac{R_{3}}{R_{2}+2R_{3}+R_{4}}$	$-\frac{{\left(A^{N}-B^{L}\right)}^{2}}{(A^{N}-B^{L}+B^{N}-A^{L})(A^{N}-B^{L}-A^{M}+B^{M})+2{\left(B^{M}-A^{M}\right)}^{2}}$
2	$\frac{R_{3}}{R_{2}+2R_{3}+R_{4}}$	$1+\frac{{\left(B^{N}-A^{L}\right)}^{2}}{{\left(B^{N}-A^{L}\right)}^{2}+(B^{N}-A^{L}-B^{M}+A^{M})(A^{N}-B^{L}-B^{M}+A^{M})+{\left(B^{M}-A^{M}\right)}^{2}}$

Then a unique numerical representation of the position relation can be formed and is applicable to all scenarios. When $N(\tilde{A},\tilde{B})$ is reduced, the position of $\tilde{A}$ is more inclined to the left of $\tilde{B}$. Therefore, the inferiority of $\tilde{A}$ to $\tilde{B}$ is increased. Conversely, the position of $\tilde{A}$ is more inclined to the right of $\tilde{B}$ when $N(\tilde{A},\tilde{B})$ is increased. Similarly, the superiority of $\tilde{A}$ to $\tilde{B}$ is increased. The range of values of the $N(\tilde{A},\tilde{B})$ for each scenario tested is shown as Eq (14).

$$N(\tilde{A},\tilde{B})=\{\begin{array}{l} (-0.5,0)\;scenario\;1\\ {}[0,1]\;scenario\;3,4,5,6,7,8,9,10\\ (1,1.5)\;scenario\;2 \end{array}\}$$ (14)

Comparing the quality of multiple hub schemes of triangular fuzzy number type requires a reference point. The fuzzy mean is proposed as a reference point $\tilde{U}$, which is calculated using $N(\tilde{A},\tilde{U})$. A larger $N(\tilde{A},\tilde{U})$ value indicates that the fuzzy number is closer to the right than the fuzzy mean, making it better than the other fuzzy numbers.

**Definition 3** Let the two hub schemes of the triangular fuzzy number type be $\tilde{A}=(A^{L},A^{M},A^{N})$ and $\tilde{B}=(B^{L},B^{M},B^{N})$ respectively. Then the fuzzy average value can be defined as Eq (15).

$$\tilde{U}=[\frac{A^{L}+B^{L}}{2},\frac{A^{M}+B^{M}}{2},\frac{A^{N}+B^{N}}{2}]≜[U^{L},U^{M},U^{N}].$$ (15)

Where $\tilde{U}=\mathrm{ave}\{\tilde{A},\tilde{B}\}$ is defined as the fuzzy average of $\tilde{A}$ and $\tilde{B}$. Then $\tilde{U}$ can be used to evaluate $\tilde{A}$ and $\tilde{B}$ as follow.

(1) Calculate $N(\tilde{A},\tilde{U})$ and $N(\tilde{B},\tilde{U})$ respectively.

(2) Compare the magnitude of both values.

1) If $N(\tilde{A},\tilde{U})<N(\tilde{B},\tilde{U})$, then $\tilde{A}<\tilde{B}$.

2) If $N(\tilde{A},\tilde{U})>N(\tilde{B},\tilde{U})$, then $\tilde{A}>\tilde{B}$.

3) If $N(\tilde{A},\tilde{U})=N(\tilde{B},\tilde{U})=0.5$, then $\tilde{A}=\tilde{B}$.

Using the following steps, the index $N(\tilde{A},\tilde{B})$ can evaluate the quality of the multiple triangular fuzzy numbers.

**Step 1**: Let ${\tilde{A}}_{i}\in F(X),i=1,2,3,\ldots ,n$.

**Step 2**: Find the $\tilde{U}=ave\{{\tilde{A}}_{1},{\tilde{A}}_{2},{\tilde{A}}_{3},\ldots ,{\tilde{A}}_{n}\}$.

**Step 3**: Calculate $N({\tilde{A}}_{i},\tilde{U}),i=1,2,3,\ldots ,n$.

**Step 4**: Evaluate based on the magnitude of indicator values.

## 3. Triangular fuzzy number-oriented Genetic-Tabu Search algorithm

Section 2 finished the establishment of the model and the design of the fitness operator in the algorithm structure. In this section, to obtain high-quality hub solutions in an efficient solution time, we further design a complete heuristic algorithm based on the proposed fuzzy NSUMApHMP. A Genetic-Tabu Search algorithm is proposed. It generates superior initial domain space using the Genetic Algorithm, and then the optimal hub scheme is obtained through iterative optimization by the global search capability of the Tabu Search algorithm.

### 3.1. Generating initial solution

The Tabu Search (TS) algorithm is well-known for its local search capabilities and memory functions. The quality of the initial solution can significantly affect its performance, particularly in large-scale problems. The convergence speed of TS algorithm is slow when the initial solution is sub-optimal, which limits its practical applicability to real-world problems.

To improve the efficiency of TS algorithm in solving large-scale problems, a hybrid strategy of Genetic-Tabu Search algorithm, which combines the advantages of Genetic Algorithm and Tabu Search algorithm, was proposed. It overcomes the shortcomings of TS in initial solutions of large-scale problems. The Genetic Algorithm is used to generate a high-quality initial solution, which are served as the input for the Tabu Search algorithm. The following pseudocode example illustrates the process of generating initial solutions.

**Algorithm 1**: Initialization based on Genetic algorithm

**Input:**

Scale (*n*), Number of hubs(*p*)

**output:**

Initial hub allocation

**while** reaching stopping conditions

/* Initialization of the population (*Hubs*)

**for** 1 to n

randomly generate a chromosome that includes the network structure (hub selection and connection)

**end for**

/* Chromosome crossover

**while** the offspring population < n

select parents using the roulette wheel selection method, swap hub locations, and then reassign connections based on the updated hub locations

/* Chromosome mutation

mutate one hub and reassign connection methods

**end while**

/* Calculate fitness value

**for** 1 to n

calculate the total network cost as the fitness of each chromosome

**end for**

**end while**

/* Return output (*Hubs*)

### 3.2. Defining the neighborhood space and its candidate solution set

Given that there are *p*(*n*−*p*) feasible solutions in the neighborhood space, all obtained neighborhood solutions are served as the candidate solution set to be generated for the next step.

### 3.3. Designing fitness operator

As mentioned above, the fitness operator is basis of the evaluation index. It can be depicted as the following pseudocode example. In addition, the fitness operator will be also used in the Floyd-Warshall algorithm to solve the shortest path problem for node connectivity.

**Algorithm 2**: Computation of the fitness operator

**Input:**

size of the candidate solution set (*m*),cost parameters of solution *i* in the candidate solution set $\left({\tilde{A}}_{i}\right)$, fuzzy average of the objective function values $\left(\tilde{U}\right)$,

**output:**

evaluating results of the solution (*rank*_*i*_)

/* Conduct evaluation

**for** *i* in *m*

**if** ${A_{i}}^{L}\leq U^{L}$

 **if** ${A_{i}}^{M}\leq U^{M}$

  **if** ${A_{i}}^{N}\leq U^{N}$

   **if** ${A_{i}}^{N}\leq U^{L}$, then $rank_{i}=N_{1}({\tilde{A}}_{i},\tilde{U})$ **else** $rank_{i}=N_{3}({\tilde{A}}_{i},\tilde{U})$

  **else** $rank_{i}=N_{5}({\tilde{A}}_{i},\tilde{U})$

 **else**

  **if** ${A_{i}}^{N}\leq U^{N}$, then $rank_{i}=N_{4}({\tilde{A}}_{i},\tilde{U})$ **else** $rank_{i}=N_{6}({\tilde{A}}_{i},\tilde{U})$

**else**

 **if** ${A_{i}}^{M}\leq U^{M}$

  **if** ${A_{i}}^{N}\leq U^{N}$, then $rank_{i}=N_{7}({\tilde{A}}_{i},\tilde{U})$ **else** $rank_{i}=N_{9}({\tilde{A}}_{i},\tilde{U})$

 **else**

  **if** ${A_{i}}^{N}\leq U^{N}$, then $rank_{i}=N_{8}({\tilde{A}}_{i},\tilde{U})$

  **else**

   **if** ${A_{i}}^{L}\geq U^{N}$, then $rank_{i}=N_{2}({\tilde{A}}_{i},\tilde{U})$ **else** $rank_{i}=N_{10}({\tilde{A}}_{i},\tilde{U})$

**end for**

/* Return output *rank*_*i*_

### 3.4. Determining Tabu list and length

To minimize the roundabout search, the Tabu list follows the First-In-First-Out (FIFO) principle and uses the fixed setting method to determine the Tabu length. Each exchanged hub node is given a Tabu length and then is added to the Tabu list. It can be released only if its Tabu length equals zero or if it satisfies the aspiration criterion.

### 3.5. Setting aspiration criterion

The aspiration criterion adopted in this paper includes two aspects.

(1) If a Tabu candidate solution is better than the current optimal solution, it is updated with the current solution and the optimal solution.

(2) If all candidate solutions are Tabu and are inferior to the current optimal solution, then the optimal Tabu candidate solution is updated to the current solution.

### 3.6. Procedure of Genetic-Tabu Search algorithm

**Fig 11:**
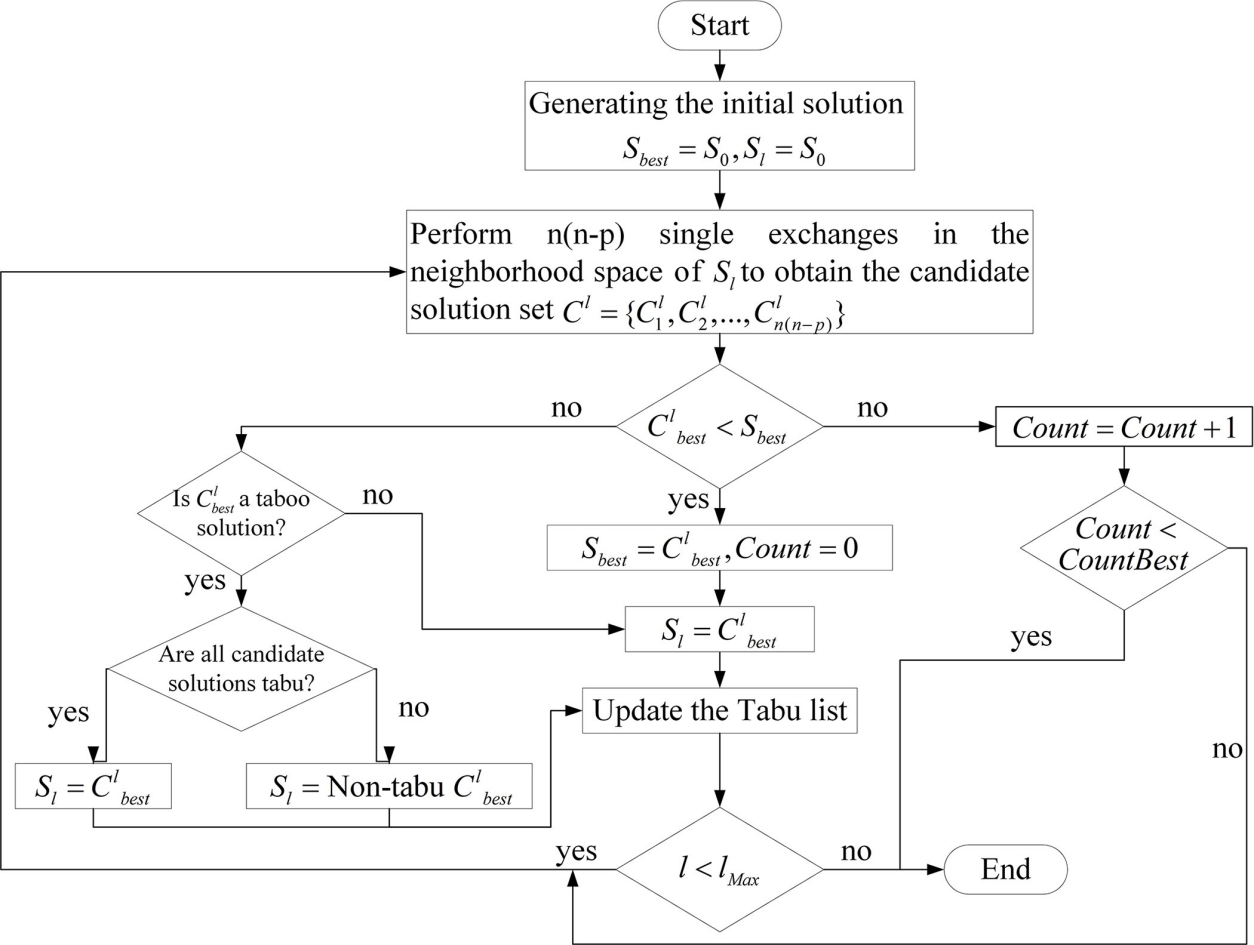
The procedure of the Genetic-Tabu Search algorithm.

By inputting the passenger demand volume parameter and unit flow cost parameter of the triangular fuzzy number type into the Genetic-Tabu Search algorithm, the optimal hub solution is obtained through iteration of the algorithm process. The whole procedure of the Genetic-Tabu Search algorithm can be depicted in Fig 11, where *S*_*best*_ is the optimal solution, *l* is the number of iterations, *l*_*Max*_ is the maximum number of iterations, *S*_0_ is the initial solution, *S*_*l*_ is the current solution, $C_{best}^{l}$ is the best solution among the current candidate solutions, and *CountBest* is the maximum number of times to keep the optimal solution.

As shown in Fig 11, the algorithm begins by generating an initial solution using a Genetic Algorithm. In each iteration, for each non-hub node and each hub node in the set, individual exchanges are performed. Among these exchanges, the algorithm selects the optimal candidate solution. If this candidate solution is superior to the current best solution, the solution updates. Otherwise, the solution is checked for Tabu status. If all candidate solutions are Tabu, the best Tabu solution is chosen as the current solution. After an iteration, the algorithm updates the current best solution, the current solution, and the Tabu list. Subsequently, it checks whether the stopping criteria are met. If they are, the results are output, and the algorithm terminates. Otherwise, the process continues with further exchanges and updates until the stopping criteria are satisfied.

## 4. Numerical example

To verify the effectiveness of the customized algorithm and the proposed approach in an uncertain environment in this research, we conducted numerical example calculation experiments. In this section, the Civil Aeronautics Board data set (CAB) introduced by O’Kelly [2] and several self-constructed data sets are used to verify the proposed optimization approach and customized algorithm. The experiment is implemented under a computer environment with Windows 10, 16G of computing memory, and 2.6GHz CPU, using Java JDK version 1.8. The Tabu length, the stopping condition, and the maximum number of iterations are set to 10, 50, and 1000, respectively. To test the algorithm’s effectiveness in solving large-scale problems, the CAB is used to randomly generate a network with 200 nodes, and the original 25 nodes with real data (called for self-constructed data set for simplicity) were selected to verify the reliability of the model.

### 4.1. Data generation and its illustration

Self-constructed data set and triangular fuzzy number-based parameters for unit flow cost and demand volume are generated using the CAB data set. The dataset’s real number data are set as the most probable values *A*^*M*^ in numerical experiments. A method is developed to generate all related parameter values in the proposed model, ensuring randomness and representing all overlapping scenarios. Additionally, a method is developed to generate probability distributions conforming to triangular fuzzy numbers for stochastic simulation experiments.

(1) Generation of self-constructed data set

To evaluate the performance of the algorithm on a large scale, the 25 city nodes from the CAB dataset were taken as the basic nodes and the CAB dataset expanded to a self-constructed data set with 200 nodes. Subsequently, 175 unique virtual nodes were randomly generated within a 2000×2000 grid to ensure that the triangular inequality holds between all nodes. The unit flow cost of the generated virtual nodes is proportional to their distance from other nodes, while the corresponding passenger flow is randomly generated within the range of the minimum to maximum demand volume from the original CAB dataset.

(2) Generation of triangular fuzzy number parameters

The demand volume $\tilde{W}$ and unit flow cost $\tilde{C}$ are assumed to yield normal distribution and are independent of each other. The data in the CAB data set are taken as the mean *μ* and the variance is set to *βμ*(*β*<1). To investigate the variation of the hub solution when the demand volume and unit flow cost are changed, data sets with narrow intervals and wide intervals are designed. The specific randomly generated data format is shown in Table 3.

**Table 3:** Parameters in the form of triangular fuzzy numbers.

Parameter	Interval	Triangular fuzzy number		
		Lower bound generation range *A*^*L*^	Most probable value generation range *A*^*M*^	Upper bound generation range *A*^*N*^
demand volume $\tilde{W}$	narrow	(0,*μ*)	(*A*^*L*^,*A*^*N*^)	$\left(\mu ,\mu +3\sqrt{(1/6)\mu }\right)$
	wide	(0,*μ*)	(*A*^*L*^,*A*^*N*^)	$\left(\mu ,\mu +3\sqrt{(3/5)\mu }\right)$
unit flow cost $\tilde{C}$	narrow	(0,*μ*)	(*A*^*L*^,*A*^*N*^)	$\left(\mu ,\mu +3\sqrt{(1/6)\mu }\right)$
	wide	(0,*μ*)	(*A*^*L*^,*A*^*N*^)	$\left(\mu ,\mu +3\sqrt{(3/5)\mu }\right)$

(3) Data generation method for simulation experiments

The real number data for the simulation experiments should to satisfy the triangular fuzzy number probability distribution. Here, the probability density function of the triangular fuzzy number $\tilde{A}$ by 0.5(*A*^*L*^,*A*^*N*^) is calculated with the membership function in Eq (9) in Section 2.

$$f_{\tilde{A}}(x)=\{\begin{array}{l} \frac{2(x-A^{L})}{\left(A^{M}-A^{L})(A^{N}-A^{L}\right)}\;A^{L}\leq x\leq A^{M}\\ \frac{2(A^{N}-x)}{\left(A^{N}-A^{M})(A^{N}-A^{L}\right)}\;A^{M}\leq x\leq A^{N}\\ \;0\;otherwise \end{array}$$ (16)

Transforming Eq (16) into a probability density function, and then we use the inverse transformation method to obtain the stochastic simulation formula Eq (17) for variable *x*. *r* is a random number that yields [0, 1] uniform distribution.


$$f_{\tilde{A}}(x)=\{\begin{array}{l} A^{L}+{\left[r(A^{M}-A^{L})(A^{N}-A^{L})\right]}^{0.5}\;r\leq (A^{M}-A^{L})(A^{N}-A^{L})\\ A^{N}-{\left[(1-r)(A^{N}-A^{M})(A^{N}-A^{L})\right]}^{0.5}\;r\leq (A^{M}-A^{L})(A^{N}-A^{L}) \end{array}$$ (17)

### 4.2. The effectiveness of the algorithm

To evaluate the effectiveness of the proposed customized algorithm in solving the NSUMApHMP, the CPU time and accuracy of the proposed algorithm are compared with other algorithms on the self-constructed data set, including the Genetic Algorithm (GA) from Qin Z [33], the Tabu Search algorithm (TS), and the Branch-and-Bound algorithm (B&B) in the GUROBI solver. GTS, TS, and GA are compared with symmetric triangular fuzzy numbers and real numbers generated from the self-constructed data set. Since the GUROBI solver cannot directly utilize fuzzy numbers, it can only perform calculations using the real numbers generated from the self-constructed data set. The experimental instances were set with a range of 10 to 200 nodes, encompassing 11 different scales, and a discount factor *α* of 0.8 was applied. In addition, for the parameter settings of GA, the experiments are set with a population size of 400, a crossover probability of 0.2, probabilities of 0.05 and 0.3 for the hub and linkage condition group variants respectively, a discount factor *α* = 0.8, and the stopping conditions equal to the values in GTS.

The accuracy of all algorithms was compared using GAP as shown in Eq (18).

$$GAP=\frac{\left|UB-LB\right|}{UB}*100\%$$ (18)

For GTS, TS, and GA in real number data, their UBs are the upper bounds obtained by the GUROBI solver. Their LBs is the optimal objective function value. For GTS, TS, and GA in triangular fuzzy number data, their UBs are the upper bounds obtained by the GTS. The results for real number data and triangular fuzzy number data are displayed in Tables 4 and 5, respectively.

**Table 4:** Efficiency comparison of GTS, TS, GA, and B&B in real number data.

Instance	GTS		TS		GA		B&B	
	GAP(%)	CPU Time (s)	GAP(%)	CPU Time (s)	GAP(%)	CPU Time (s)	GAP(%)	CPU Time (s)
**10–2**	0.00	0.05	0.00	0.08	0.00	1.04	0.00	0.02
**20–3**	0.00	0.31	0.00	0.46	0.00	2.12	0.00	6.39
**40–4**	0.47	1.75	0.42	2.01	1.16	3.69	0.23	786.76
**60–5**	1.69	8.98	1.27	16.13	2.13	23.62	-	-
**80–6**	1.53	34.95	1.46	69.21	2.30	86.80	-	-
**100–7**	2.20	104.06	2.58	199.40	2.57	259.87	-	-
**120–8**	2.27	224.42	3.90	413.07	2.49	542.72	-	-
**140–9**	3.06	549.50	4.37	842.91	3.73	1168.16	-	-
**160–10**	3.35	1082.90	4.55	1793.35	4.97	2021.54	-	-
**180–11**	3.46	2295.62	5.48	3510.92	5.42	4331.45	-	-
**200–12**	5.01	4171.89	6.14	7294.27	5.68	8192.64	-	-
**AVE**	2.09	770.40	2.79	1285.62	2.77	1512.15	-	-

Note. “-” = The solution cannot be obtained within 3 hours.

**Table 5:** Efficiency comparison of GTS, TS, GA, and B&B in triangular fuzzy number data.

Instance	GTS		TS		GA		B&B	
	GAP(%)	CPU Time (s)	GAP(%)	CPU Time (s)	GAP(%)	CPU Time (s)	GAP(%)	CPU Time (s)
**10–2**	0.00	0.06	0.00	0.10	0.00	1.24	-	-
**20–3**	0.00	0.34	0.00	0.53	0.00	2.52	-	-
**40–4**	0.00	1.97	0.15	2.33	1.01	4.31	-	-
**60–5**	0.00	9.92	0.38	19.01	1.77	28.32	-	-
**80–6**	0.00	38.52	0.66	80.71	1.45	104.05	-	-
**100–7**	0.00	119.32	1.18	235.45	2.43	303.14	-	-
**120–8**	0.00	254.67	1.59	492.09	3.03	638.02	-	-
**140–9**	0.00	623.79	1.45	993.03	3.27	1363.59	-	-
**160–10**	0.00	1223.68	1.98	2078.13	3.58	2375.11	-	-
**180–11**	0.00	2597.72	1.78	4073.02	3.66	5196.87	-	-
**200–12**	0.00	4731.34	2.30	8531.38	3.89	9633.73	-	-
**AVE**	0.00	872.85	1.04	1500.53	2.46	1786.45		

Note. “-” = The solution cannot be obtained within 3 hours.

Based on the performance of various algorithms using the real-number data in Table 4, it can be observed that the B&B algorithm can only obtain effective solutions for up to 40 nodes within 3 hours. From the perspective of CPU time, for the same problem size, GA requires the longest CPU time, followed by TS, while the average CPU time of GTS is 49.05% and 40.93% shorter than that of GA and TS, respectively. The improvement of the GTS algorithm lies in its enhanced utilization of the advantages of the Genetic Algorithm during the search process. In terms of the solution accuracy measure, TS can maintain the lowest GAP for small-scale instances with nodes less than 80, attributed to its characteristics of neighborhood search. With the increase in scale, the average GAP for TS and GA became 2.79% and 2.77%, respectively. In comparison, GTS, utilizing GA to generate high-quality initial solutions and using them as the initial neighborhood for search, converges to superior solutions during problem-solving. It results in lower GAP of 2.09% in large-scale instances. This insight suggests that, in practical applications, particularly for problems involving a larger number of nodes, the integration of various algorithmic strengths can be employed to enhance computational efficiency.

Table 5 presents the results of calculations using triangular fuzzy numbers, and it is evident that B&B algorithm cannot solve them due to its nature as an exact algorithm. By comparing the average CPU time of the other three algorithms in Table 4, the average CPU time in Table 5 of GTS, TS, and GA increased by 11.74%, 14.32%, and 15.35% respectively. This indicates that although introducing a triangular fuzzy number evaluation index can better express uncertainty, it also comes with a certain computational cost. Nevertheless, as shown in Table 5, GTS continues to maintain faster computation time and higher computational accuracy, with a CPU time of 872.85s and the lowest GAP.

### 4.3. The effectiveness of the optimization approach under uncertainty

To validate the effectiveness of the proposed optimization approach (Approach 1) in an uncertain environment, we compared it with three other classical approaches: a stochastic optimization approach (Approach 2) [14], a robust optimization approach (Approach 3) [34], and an approach based on real-point data types [4] (Approach 4). First, we generated passenger demand and unit flow cost parameters based on the triangular fuzzy number data in Table 3. Next, the parameters were used to solve the hub schemes for all four approaches. Approach 1 employed a customized algorithm, while the other approaches used the GUROBI solver. Subsequently, as described in Section 5.1 (Method (2)), the generated real-type parameters for passenger demand and unit flow cost were applied in a simulation experiment. The specific steps of the simulation experiment involved randomly generated real values for passenger demand volume and the unit flow cost, which are within the specified range of the generated triangular fuzzy number parameters. These real parameters were then substituted into the hub schemes of the four approaches to calculate the corresponding total cost. Through simulation experiments, the adaptability of the four approaches in uncertain environments can be compared.

In this experimental section, the other parameters were set as the narrow interval and the wide interval condition: the scale was set to 10, 15, 20, and 25, the number of hubs was set to 3, 4, and 5, and the discount factor was set to 0.4 and 0.8. The obtained hub schemes by the four approaches under different instances with given uncertain parameters are presented in Tables 6 and 7.

**Table 6:** Simulation results for a discount factor of 0.8.

Instance	Hub Scheme (narrow interval)				Hub Scheme (wide interval)			
	Approach 1	Approach 2	Approach 3	Approach 4	Approach 1	Approach 2	Approach 3	Approach 4
**10–3**	2,4,5	3,4,8	3,4,8	3,6,7	3,6,8	3,6,8	3,6,8	3,6,7
**10–4**	3,4,5,7	3,4,6,8	3,4,6,8	2,3,6,7	2,3,6,8	0,3,6,8	3,6,7,8	2,3,6,7
**10–5**	0,3,4,5,7	3,4,6,7,8	3,4,6,7,8	2,3,5,6,7	0,2,3,6,8	0,2,3,6,8	1,3,6,7,8	2,3,5,6,7
**15–3**	7,11,12	5,11,12	5,7,12	3,6,11	5,10,11	3,5,10	5,6,11	3,6,11
**15–4**	7,8,11,12	5,6,11,12	5,7,11,12	3,6,7,11	5,6,10,11	0,3,5,10	3,5,6,11	3,6,7,11
**15–5**	3,5,6,11,12	3,5,6,11,12	5,7,10,11,12	3,6,7,11,13	0,5,6,10,11	0,3,5,6,10	3,5,6,11,12	3,6,7,11,13
**20–3**	4,12,18	1,4,12	1,4,11	3,11,16	4,8,17	8,10,17	7,8,17	3,11,16
**20–4**	0,4,12,18	1,4,6,17	1,3,4,11	3,11,13,16	4,8,13,17	1,7,8,17	3,7,8,17	3,11,13,16
**20–5**	0,1,4,12,18	1,4,6,11,17	1,3,4,6,11	3,6,11,13,16	1,4,7,8,18	1,3,7,8,17	3,7,8,13,17	3,6,11,13,16
**25–3**	8,11,24	4,7,8	7,8,17	3,11,16	1,10,19	10,17,20	10,20,24	3,11,16
**25–4**	4,8,11,24	1,4,7,8	7,8,16,23	3,11,13,16	1,5,10,19	5,10,17,20	5,17,20,24	3,11,13,16
**25–5**	1,4,8,11,24	1,4,7,8,17	5,7,8,16,23	3,11,13,16,21	0,11,17,20,24	5,10,17,20,23	11,17,20,23,24	3,11,13,16,21

**Table 7:** Simulation results for a discount factor of 0.4.

Instance	Hub Scheme (narrow interval)				Hub Scheme (wide interval)			
	Approach 1	Approach 2	Approach 3	Approach 4	Approach 1	Approach 2	Approach 3	Approach 4
**10–3**	4,6,7	3,4,8	3,6,8	3,6,7	2,3,6	3,6,8	2,6,8	3,6,7
**10–4**	3,5,6,7	3,4,7,8	3,6,7,8	2,3,6,7	2,3,6,8	2,3,6,8	2,3,6,8	2,3,6,7
**10–5**	2,3,5,6,7	3,4,6,7,8	2,3,6,7,8	2,3,6,7,8	2,3,6,7,8	2,3,6,7,8	2,3,6,7,8	2,3,6,7,8
**15–3**	8,11,12	5,11,12	5,11,12	3,6,11	5,10,11	3,6,11	5,6,11	3,6,11
**15–4**	3,5,11,12	3,5,11,12	5,6,11,12	3,6,11,13	5,6,11,13	0,3,6,11	5,6,11,13	3,6,11,13
**15–5**	3,5,6,11,13	3,5,6,11,12	3,5,6,11,13	2,3,6,11,13	3,5,6,11,13	3,5,6,11,13	3,5,6,11,13	2,3,6,11,13
**20–3**	4,11,16	4,11,16	4,11,16	3,11,16	8,11,17	8,11,17	8,11,13	3,11,16
**20–4**	4,11,13,16	4,11,13,16	4,11,13,16	3,11,13,16	8,11,13,17	8,11,13,17	3,11,13,16	3,11,13,16
**20–5**	3,4,11,13,16	3,4,11,13,16	4,6,11,13,16	3,6,11,13,16	6,8,11,13,16	6,8,11,13,17	6,8,11,13,16	3,6,11,13,16
**25–3**	4,11,16	4,11,17	11,16,20	3,11,16	3,11,24	3,11,17	10,11,24	3,11,16
**25–4**	8,11,16,23	4,11,16,23	11,13,16,20	3,11,13,16	8,11,16,23	3,11,13,17	3,11,13,24,	3,11,13,16
**25–5**	1,8,11,23,24	4,8,11,16,23	7,8,11,16,23	3,6,11,13,16	1,8,11,23,24	3,6,11,13,17	3,6,11,13,24	3,6,11,13,16

Based on the observations in Tables 6 and 7, we can reveal the following observations.

(1) Under the same conditions, the hub solutions of the four approaches vary with different scales. As the scale increases, the differences in hub solutions among the four methods become more significant. This implies that in real-world aviation operations, as the scale of the problem expands and demand within the network increases, it leads to different layouts for real aviation hubs.

(2) When the discount factor changes, the hub solutions of the four approaches change. This indicates that the discount factor significantly influences the decision outcomes. The reason is that changes in the discount factor led to different trade-offs in transportation costs, affecting the selection of real aviation hub locations and the distribution of network traffic.

(3) When the width of the interval changes, the hub solutions of the first three approaches, which consider uncertainty, change. Since Approach 4 does not consider uncertainty, the hub solutions remain unchanged. This suggests that approaches considering uncertainty can more flexibly adapt to changes in the real aviation operational environment.

**Fig 12:**
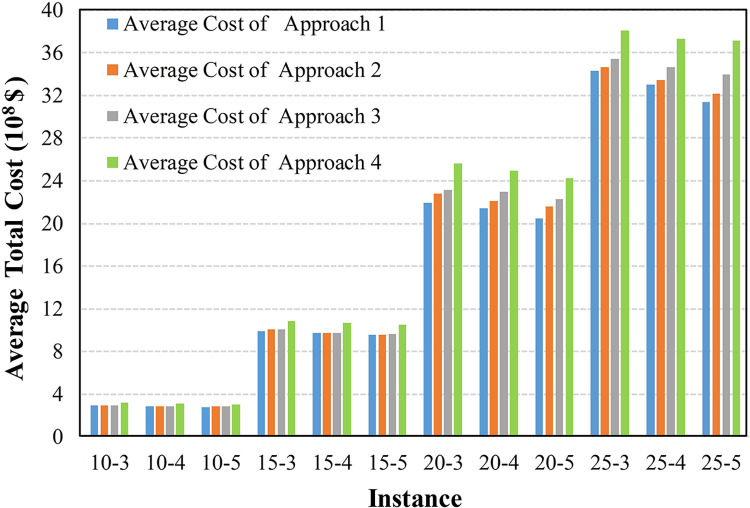
The average cost with a discount factor of 0.8 and a narrow interval.

**Fig 13:**
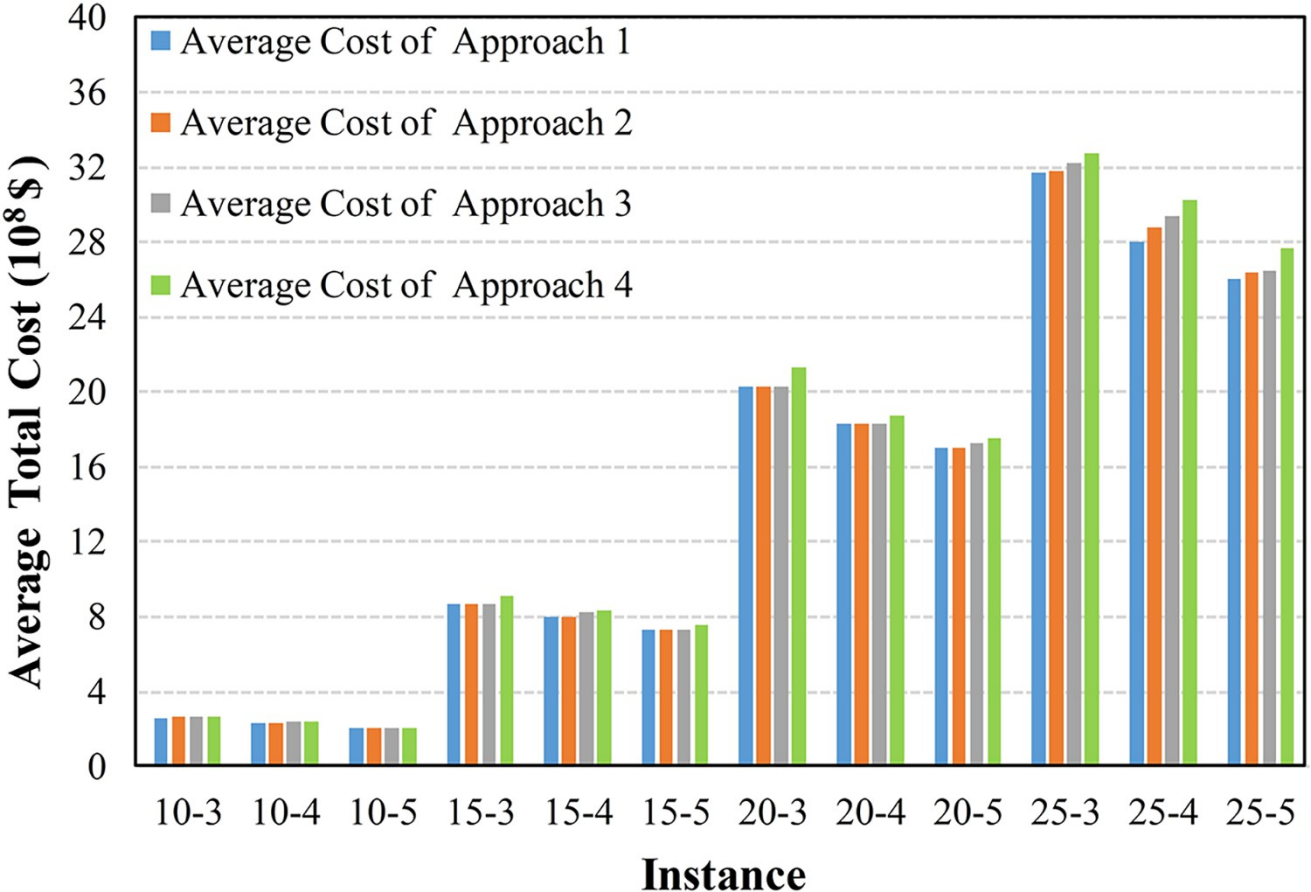
The average cost with a discount factor of 0.8 and a wide interval.

**Fig 14:**
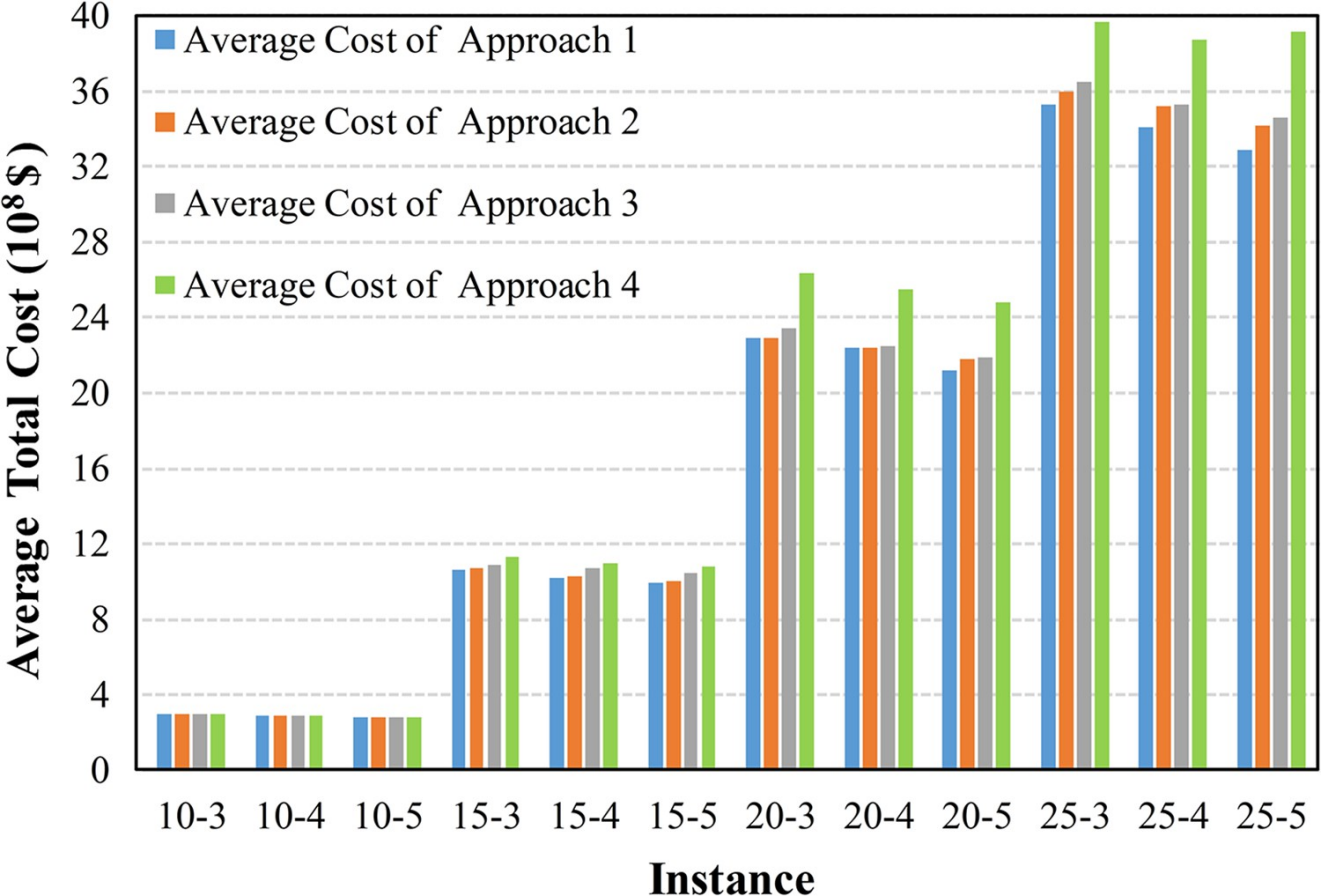
The average cost with a discount factor of 0.4 and a narrow interval.

**Fig 15:**
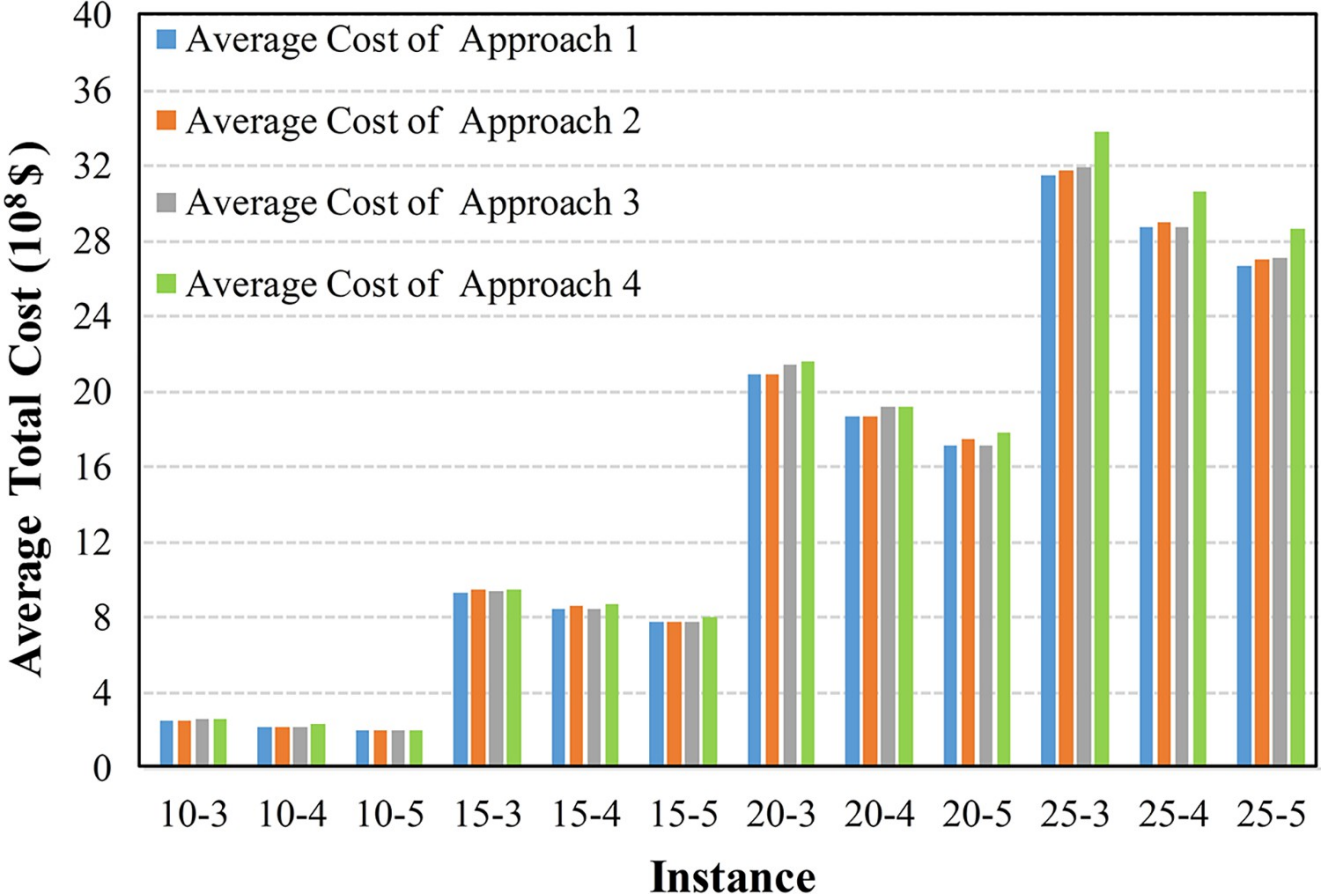
The average cost with a discount factor of 0.4 and a wide interval.

To further compare the adaptability of these approaches in uncertain environments, Figs 12–15 display the average cost of their generated solutions over 1000 simulation experiments.

The results in Figs 12–14 indicate that Approach 4, which does not consider any uncertainty, exhibits the highest average total cost in each scenario, indicating its inferior adaptability in uncertain environments. Approaches 2 and 3, employing random optimization and robust optimization, respectively, show a reduced average total cost compared with Approach 4. In most cases, Approach 1, incorporating the triangular fuzzy number evaluation index, provides the lowest average total cost solution. This is because Approaches 2 and 3 can only utilize information limited to several discrete and finite scenarios. In contrast, Approach 1, employed the proposed evaluation index and utilized continuous uncertain parameters represented by triangular fuzzy numbers.

Furthermore, variations in the discount factor also impact the hub selection of the four approaches. A detailed analysis of the fluctuations in hub schemes (changes in hub nodes) under different discount factor conditions for the four approaches is presented in Table 8, comparing the specific changes in hub schemes between Tables 6 and 7. The hub configuration of Approaches 1 to 4 changed 48,39,40 and 8 times, respectively. This indicates that the evaluation index of our approach in this study can more accurately balance changes in transportation cost within the aviation hub network. It also suggests that our approach in this study is better suited to cope with external environmental fluctuations.

**Table 8:** Fluctuation of hub schemes for four approaches.

Approach type	Number of changes in nodes in the hub schemes
**Approach 1**	48
**Approach 2**	39
**Approach 3**	40
**Approach 4**	8

The above results imply that, in addressing the *p*-hub median problem in the aviation transportation industry, our optimization approach, which considers continuous uncertainty factors, can offer superior and robust solutions. It provides a more comprehensive modeling of uncertainty, enabling better adaptation to changes in uncertainty levels and discount factors. Consequently, it aids in optimizing the location of aviation hubs and the allocation of traffic, enhancing operational efficiency, and reducing cost.

### 4.4. Sensitivity analysis

To further investigate how the uncertainty level of triangular fuzzy number parameters affects the total transportation cost of hub solutions, this section examines the proposed optimization approach in obtaining solutions for NSUMApHMP in an uncertain environment. The uncertainty levels of passenger demand volume and unit flow cost are reflected by variations in the interval width and the most probable values, with a discount factor of 0.8. The specific results of the hub scheme are provided in Tables A1 to A5 in S1 Appendix.

#### 4.4.1. Impact of interval width

(1) When the most probable value remains unchanged, increase the interval width

**Fig 16:**
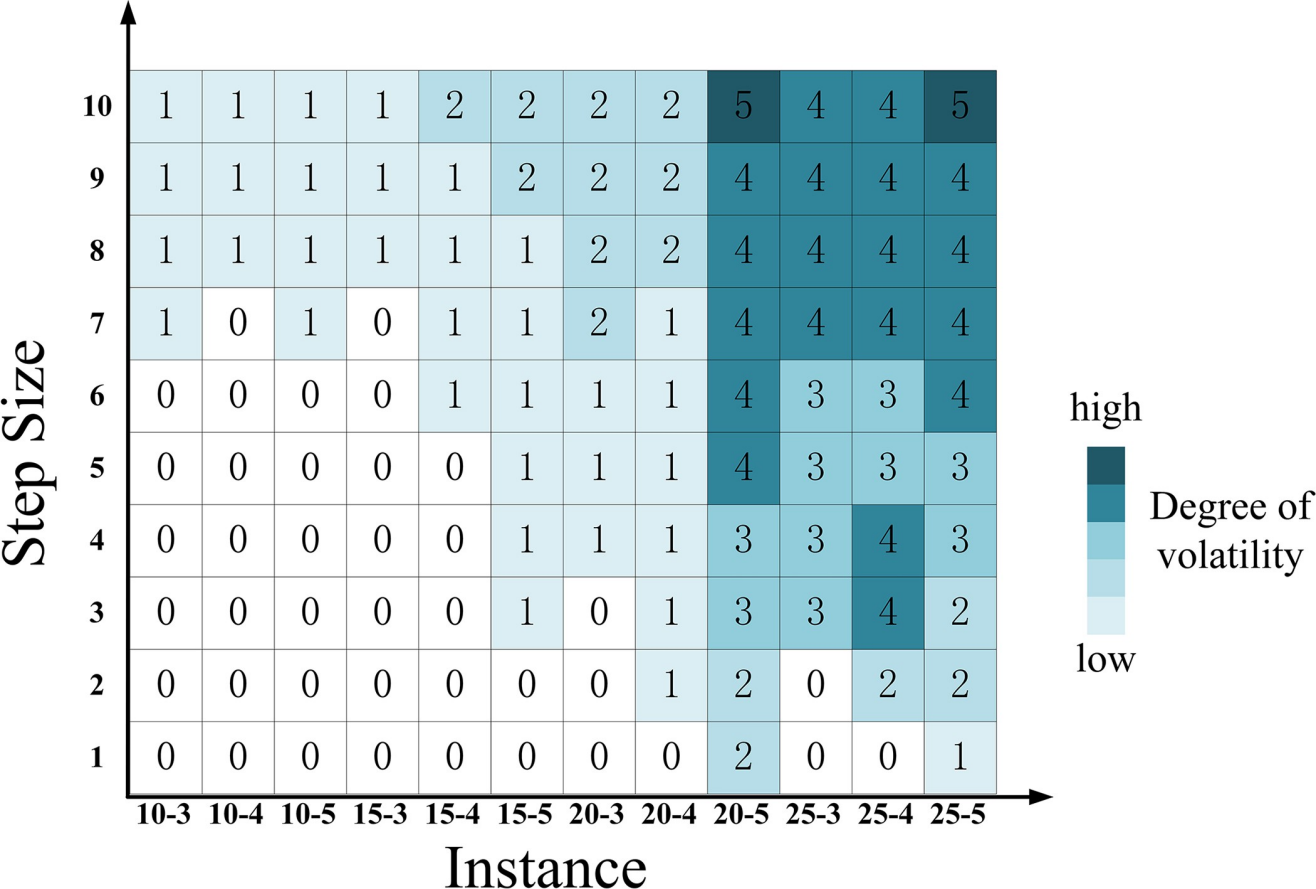
The fluctuation of the hub scheme impacted by increasing the interval width alone.

As shown in Fig 16, the lower bound of the parameters gradually moves towards zero, while the upper bound increases up to 1.2 times the original upper bound, with a step size of one-tenth of 0.2*A*^*R*^. The lower bound step size is one-eleventh of *A*^*L*^ to ensure the effectiveness of the Floyd-Warshall algorithm. The upper and lower bound of the interval are simultaneously expanded outward by 10 steps. Hub scheme differences between each step and the original scheme are compared to obtain the hub fluctuation.

Fig 16 illustrates that, with the increase in interval width, the disparity between the initial hub configuration and the outcome in NSUMApHMP grows, indicating an escalation in problem uncertainty. This phenomenon is attributed to alterations in the relative positioning of the membership functions of triangular fuzzy number parameters, leading to variations in hub schemes. Additionally, as the scale increases, the extent of change in the hub schemes for a network with 25 nodes (depicted by the deep segment in Fig 16) is noticeably greater than that of networks with 10, 15, and 20 nodes (depicted by the light segments in Fig 16). This indicates a positive correlation between the impact of interval width on the hub scheme and the size of the network. In computations involving large-scale *p*-hub median problems, changes in interval width are more likely to impact hub scheme choices.

(2) When the most probable value remains unchanged, narrows the interval width

**Fig 17:**
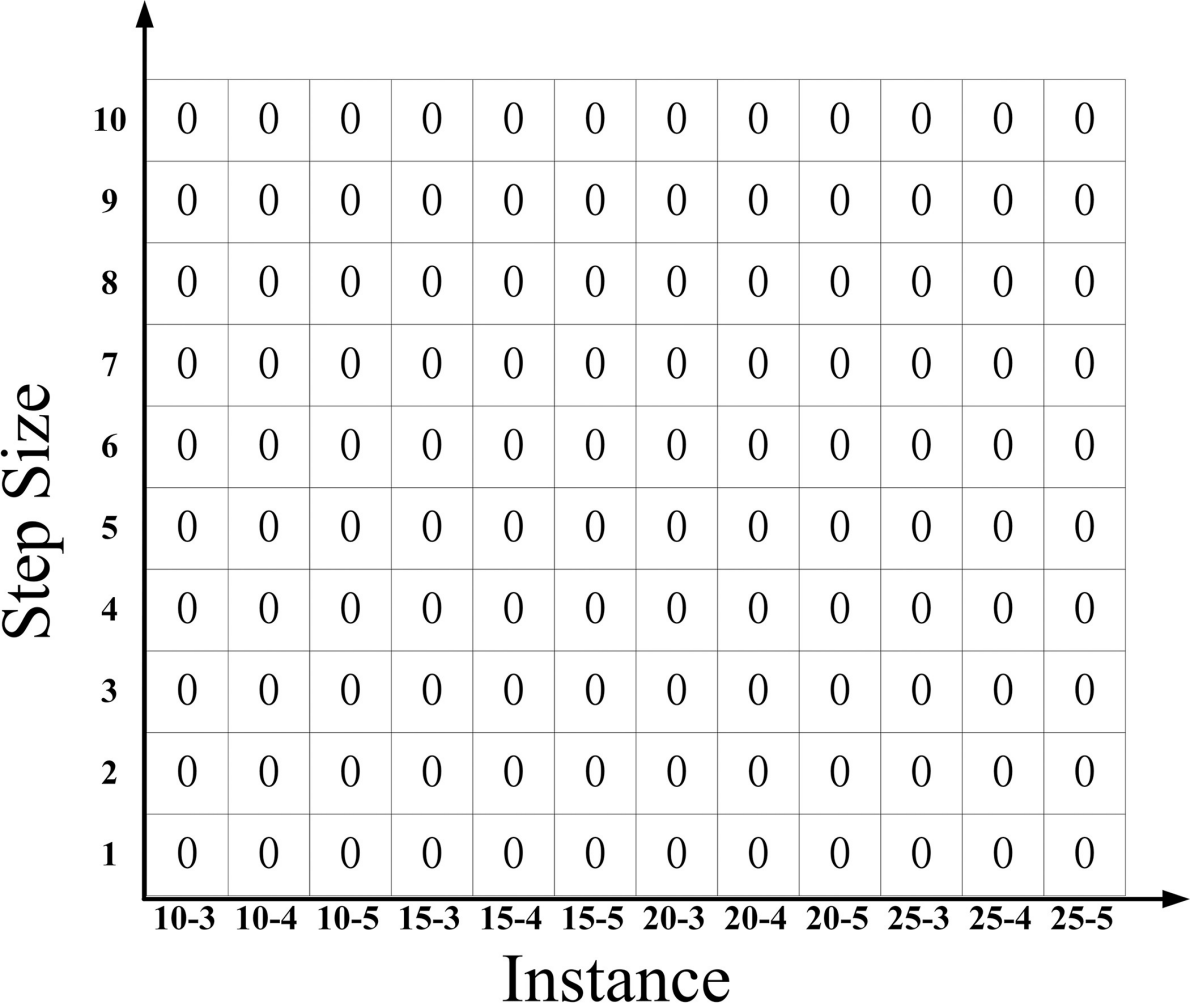
The fluctuation of the hub scheme impacted by narrowing the interval width alone.

To narrow the interval, the lower bound is adjusted to approach $A^{L}+(1/2)\times (A^{M}-A^{L})$ and the upper bound is adjusted to approach $A^{R}-(1/2)\times (A^{R}-A^{M})$, while keeping both bounds from the most probable value. The step size for the upper bound is set to one-tenth of $\left(1/2)\times (A^{R}-A^{M}\right)$ and the step size for the lower bound is set to one-eleventh of $\left(1/2)\times (A^{M}-A^{L}\right)$. The results of the variation in hub schemes are depicted in Fig 17.

According to Fig 17, it can be observed that when the interval width narrows, the hub scheme remains unchanged. This result is attributed to the fact that when the width of the parameters approaches the most probable value, the triangular fuzzy number parameters gradually transform into real numbers, leading to the reduction in the degree of uncertainty. This implies that as the range of critical parameters becomes clearer and more focused, the selection of hub schemes becomes more stable, no longer influenced by the broad range of uncertainty.

#### 4.4.2. Impact of the most probable value

**Fig 18:**
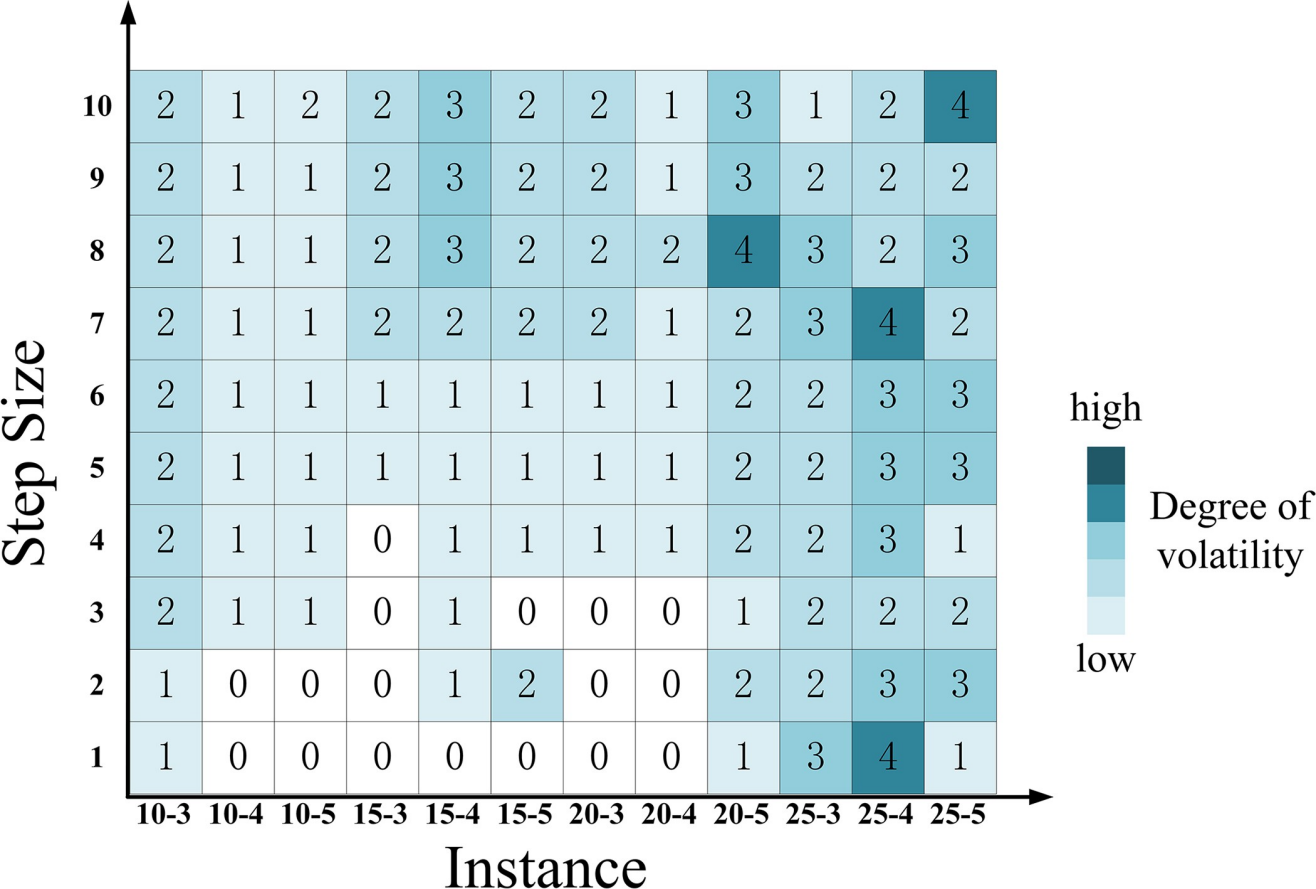
The fluctuation of the hub scheme impacted by changing the most probable value.

The lower and upper bounds for the passenger flow and cost parameters remain fixed, while the most probable value within the corresponding interval is allowed to change. To achieve this, we designed one-tenth interval width steps that move from the lower to the upper bound. The most probable value moves from the lower limit to the upper limit in increments of the specified step size. The results of the variation in hub schemes are depicted in Fig 18.

Fig 18 demonstrates that variations in the most probable values significantly impact hub schemes across the majority of scales. Sensitivity to changes in the most probable values is higher across various scales compared with the sensitivity to interval width depicted in Fig 16. This implies that, for solutions to the *p*-hub median problem, accurate estimation of the most probable values is crucial for the rational planning and operation of hubs.

#### 4.4.3. Impact of both interval width and the most probable value

(1) Change the most probable value as the interval widens.

**Fig 19:**
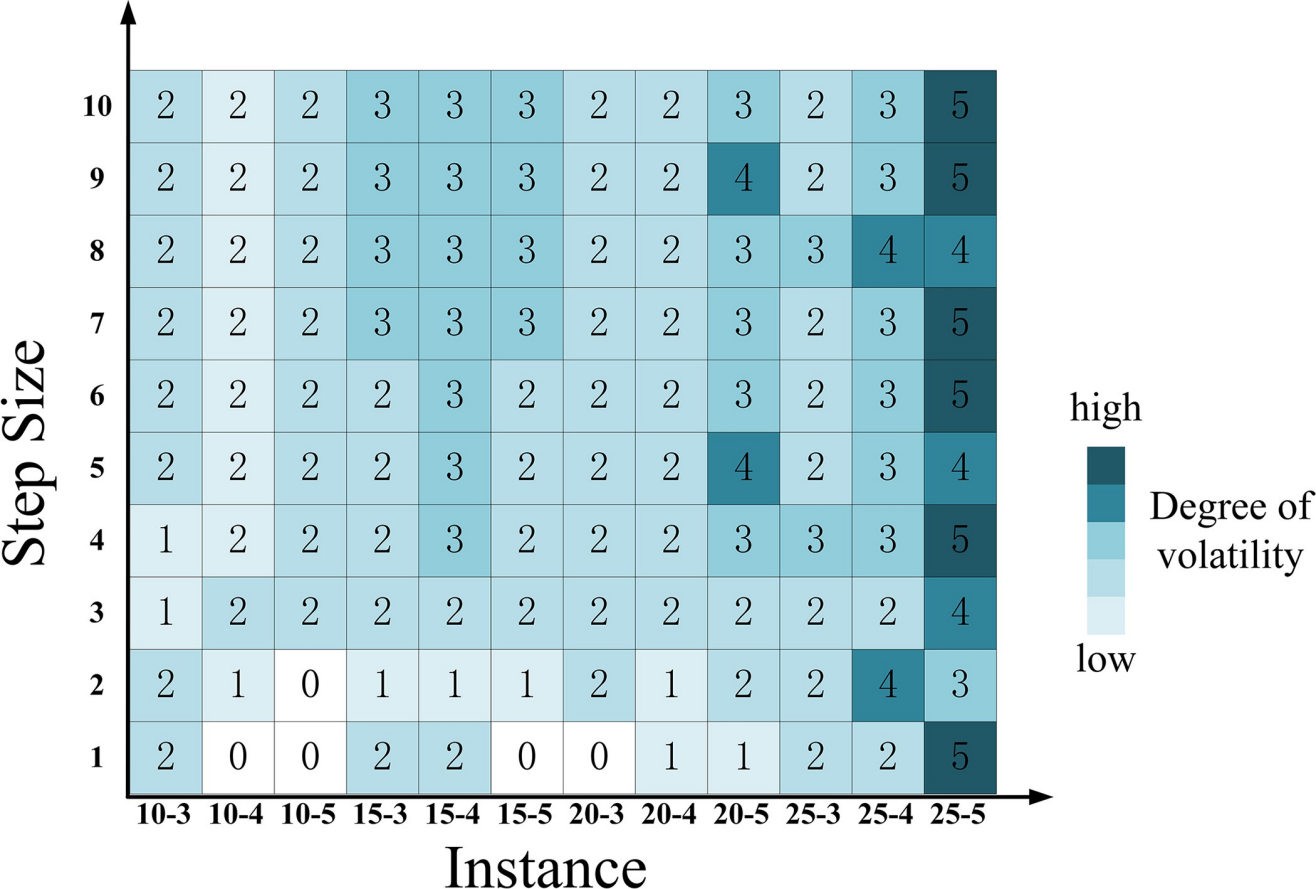
The fluctuation of the hub scheme impacted by simultaneously increasing the interval width and changing the most probable value.

Similar to the approach in Section 4.4.1, we gradually increase the interval width here. However, in this case, the most probable value is set to move within a fixed range as the interval widens. The results of the variation in hub schemes are depicted in Fig 19. In comparison to Fig 16, the hub schemes obtained in Fig 19 exhibit greater variability, especially at the scale of 25 nodes. This increased variability is attributed to the combined intensification of uncertainty in the aviation hub network represented by the most probable value and interval width. This indicates that in practical applications of addressing the *p-*hub median problem, as the uncertainty in the most probable value and interval width increases, hub schemes become more flexible and dynamic, which means greater adaptability and robustness.

(2) Change the most probable value as the interval narrows.

**Fig 20:**
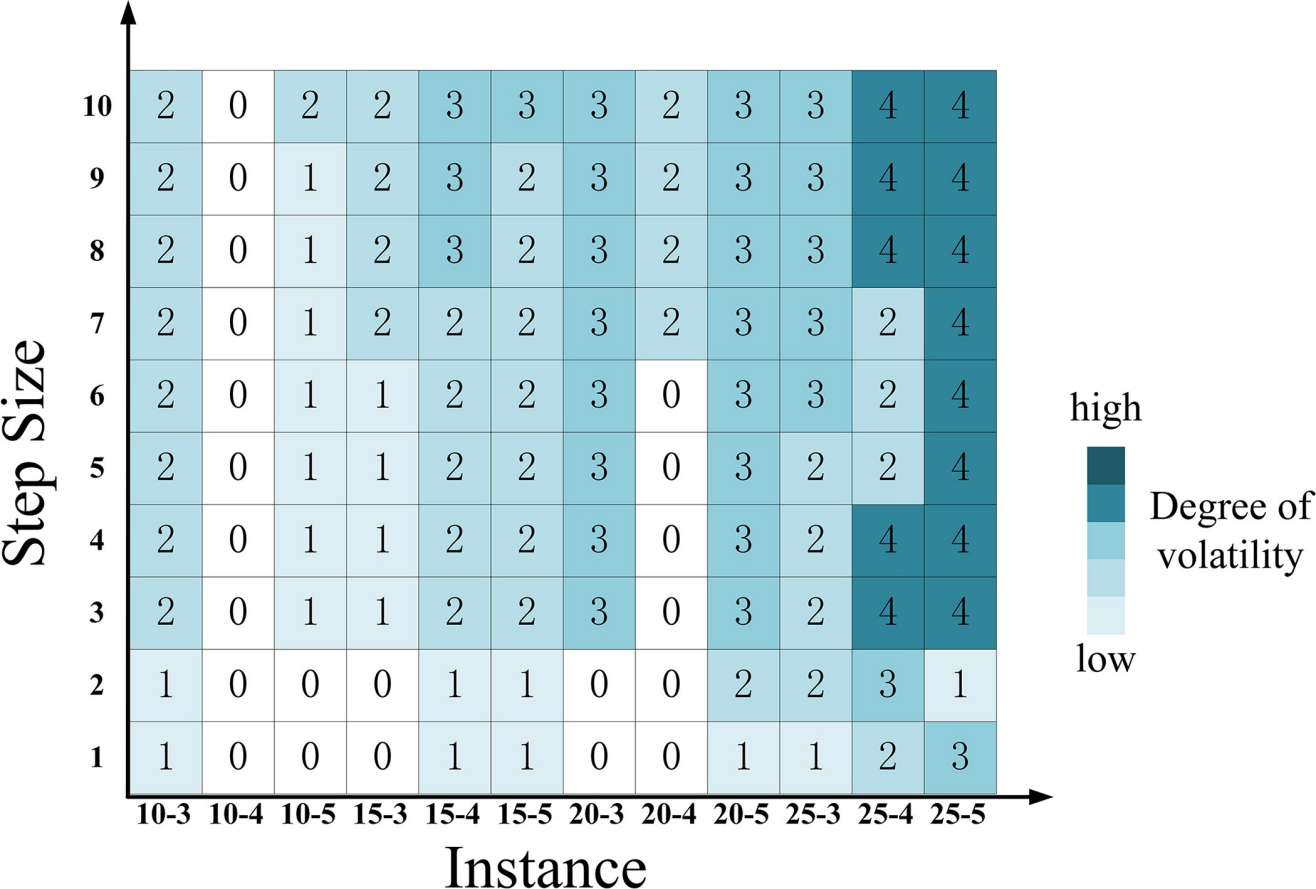
The fluctuation of the hub scheme impacted by simultaneously narrowing the interval width and changing the most probable value.

To ensure that the most probable value stays within the bound of the new interval during movement, it is set to transition from $\left(1/2)\times (A^{M}-A^{L}\right)$ to $\left(1/2)*(A^{R}-A^{M}\right)$. The range of variation for the upper bound is $\left[A^{L},A^{L}+(1/2)\times (A^{M}-A^{L})\right]$, and the range of variation for the lower bound is $\left[A^{R}-(1/2)\times (A^{R}-A^{M}),A^{R}\right]$. The resulting hub schemes generated during parameter variation are documented in Fig 20. By comparing Figs 17 and 20, we observe significant fluctuations in hub schemes when both the interval narrows and the most probable value changes simultaneously. This indicates that as data uncertainty decreases, triangular fuzzy numbers transform into real numbers, converting the fuzzy problem into a deterministic problem represented by the most probable value. Therefore, the variation in hub schemes at this point is primarily influenced by the most probable value.

## 5. Conclusions

In this study, we proposed a linear programming model based on triangular fuzzy number parameters to investigate the NSUMApHMP under uncertain conditions. To address the challenges of directly solving linear programming models with triangular fuzzy number parameters, we customized a heuristic algorithm that employs a triangular fuzzy number evaluation index as the fitness operator. To accurately evaluate the hub cost of triangular fuzzy numbers during the algorithmic iterations, we designed a ranking formula based on the membership function. To ensure both speed and precision in solving the problem, we developed a Genetic-Tabu Search algorithm, which utilizes the memorization principles of Tabu Search to find the optimal solution on top of the initial search space generated by the Genetic Algorithm. The computational results, obtained using both the classical CAB dataset and a self-constructed dataset, demonstrate the effectiveness of our proposed approach.

The simulation experiment results demonstrated that the proposed optimization approach consistently provides excellent solutions for NSUMApHMP under uncertain conditions. To validate the effectiveness of the customized algorithm, a detailed analysis was conducted. By comparing the customized algorithm with traditional TS and GA, we revealed that GTS has advantages in both solution quality and speed for resolving NSUMApHMP, as it reduced the runtime by 49.05% and 40.93%, respectively, and delivered high-quality solutions for large-scale instances. In terms of adaptability to uncertain environments, this optimization approach outperformed classical random optimization, robust optimization, and precise real-number optimization approaches, offering cost-effective and more flexible hub schemes. Sensitivity analysis indicated that both the interval width and the most probable value influence hub scheme selection. Specifically, the impact of the interval width on hub schemes was positively correlated with the scale size, while the influence of the most probable value was generally more significant across various scales than the interval width. Therefore, decision-makers should focus on anticipating and predicting changes in the most probable value and exercise effective control over the range of uncertainties, particularly as the scale gradually increases.

Despite the findings of this study on NSUMApHMP in uncertain environments, there are still some limitations that warrant further investigation. Firstly, our analysis primarily focused on hub schemes within the route network, without delving into the allocation issues of aircraft in the aviation system. However, in practical aviation operations, the unit flow cost is contingent upon the aircraft types and frequencies allocated to the routes. Simultaneously, the allocation of aircraft types and frequencies on routes is dependent on passenger demand along the origin-destination (OD) paths. This interdependency creates a mutual correlation between fleet allocation and route network design. Future research efforts could involve joint optimization of hub networks and fleet planning in uncertain environments to more comprehensively address real-world aviation transport problems. Secondly, this study relied solely on triangular fuzzy numbers to represent uncertainty. In future research, exploration of other symbolic data, such as gradient fuzzy numbers, could be considered to provide a more refined representation of uncertainty, thereby yielding a more practical decision plan.

## Supporting information

S1 Appendix The specific results of the hub scheme of Section 4.4. (DOCX)
