# Supplementary material for: A fuzzy interval optimization approach for p-hub median problem under uncertain information
Source: PLoS One. 2024 Mar 15;19(3):e0297295. doi: 10.1371/journal.pone.0297295 (PMC10942083; doi:10.1371/journal.pone.0297295)
Supplement: S1 Appendix — (DOCX) [file pone.0297295.s001.docx]

The specific results of the hub scheme of Section 4.4

**Table A1.** Results for the hub scheme with constant most probable value and wider width.

| **Instance** | **Step Size** | | | | | | | | | | |
| --- | --- | --- | --- | --- | --- | --- | --- | --- | --- | --- | --- |
|  | 0 | 1 | 2 | 3 | 4 | 5 | 6 | 7 | 8 | 9 | 10 |
| 10-3 | 3,6,8 | 3,6,8 | 3,6,8 | 3,6,7 | 3,6,7 | 3,6,7 | 2,3,8 | 3,6,7 | 3,6,7 | 3,6,7 | 3,6,7 |
| 10-4 | 2,3,6,8 | 2,3,6,8 | 2,3,6,8 | 2,3,6,8 | 2,3,6,8 | 2,3,6,8 | 2,3,6,8 | 2,3,6,8 | 2,3,6,7 | 2,3,6,7 | 2,3,6,7 |
| 10-5 | 0,2,3,6,8 | 0,2,3,6,8 | 0,2,3,6,8 | 0,2,3,4,6 | 0,2,3,4,6 | 2,3,6,7,8 | 2,3,6,7,8 | 2,3,6,7,8 | 2,3,6,7,8 | 2,3,6,7,8 | 2,3,6,7,8 |
| 15-3 | 5,10,11 | 5,10,11 | 5,10,11 | 5,10,11 | 5,10,11 | 5,10,11 | 5,10,11 | 5,10,11 | 5,10,11 | 6,10,11 | 3,6,11 |
| 15-4 | 5,6,10,11 | 5,9,10,11 | 5,6,10,11 | 5,6,10,11 | 5,6,10,11 | 5,6,10,11 | 3,5,11,13 | 3,5,11,13 | 3,5,11,13 | 3,5,11,13 | 3,5,11,13 |
| 15-5 | 0,5,6,10,11 | 5,6,7,10,11 | 5,6,7,10,11 | 5,6,7,10,11 | 5,6,7,10,11 | 3,5,6,11,13 | 3,5,6,11,13 | 3,5,6,11,13 | 3,5,6,11,13 | 3,5,6,11,13 | 3,5,6,11,13 |
| 20-3 | 4,8,17 | 4,8,17 | 8,13,17 | 8,16,17 | 1,7,18 | 1,7,18 | 8,11,17 | 8,11,17 | 8,11,17 | 8,11,17 | 8,11,17 |
| 20-4 | 4,8,13,17 | 4,8,13,17 | 4,8,13,17 | 8,11,13,17 | 8,11,13,17 | 1,3,7,18 | 8,11,13,17 | 8,11,13,17 | 3,11,13,17 | 8,11,13,17 | 8,11,13,17 |
| 20-5 | 1,4,7,8,18 | 1,3,7,13,18 | 1,3,7,13,18 | 7,8,11,13,17 | 7,8,11,13,17 | 3,8,11,13,17 | 3,8,11,13,16 | 3,8,11,13,16 | 3,8,11,13,16 | 3,8,11,13,16 | 3,6,11,13,16 |
| 25-3 | 1,10,19 | 1,10,19 | 1,10,19 | 3,7,17 | 3,7,17 | 3,7,17 | 3,7,17 | 3,7,17 | 3,7,17 | 3,7,19 | 3,7,19 |
| 25-4 | 1,5,10,19 | 1,5,10,19 | 1,10,13,19 | 3,11,13,24 | 3,11,13,24 | 3,7,19,20 | 3,5,7,13 | 3,7,8,17 | 3,7,8,17 | 3,7,8,17 | 3,7,8,17 |
| 25-5 | 0,11,17,20,24 | 0,3,11,17,24 | 0,10,11,17,24 | 0,3,11,17,24 | 0,3,11,17,24 | 0,3,11,17,24 | 0,3,11,17,24 | 0,3,11,17,24 | 0,3,11,17,24 | 5,10,11,13,17 | 5,6,7,10,18 |

**Table A2.** Results of the hub scheme with constant most probable value and narrower width.

| **Instance** | **Step Size** | | | | | | | | | | |
| --- | --- | --- | --- | --- | --- | --- | --- | --- | --- | --- | --- |
|  | 0 | 1 | 2 | 3 | 4 | 5 | 6 | 7 | 8 | 9 | 10 |
| 10-3 | 3,6,8 | 3,6,8 | 3,6,8 | 3,6,8 | 3,6,8 | 3,6,8 | 3,6,8 | 3,6,8 | 3,6,8 | 3,6,8 | 3,6,8 |
| 10-4 | 2,3,6,8 | 2,3,6,8 | 2,3,6,8 | 2,3,6,8 | 2,3,6,8 | 2,3,6,8 | 2,3,6,8 | 2,3,6,8 | 2,3,6,8 | 2,3,6,8 | 2,3,6,8 |
| 10-5 | 0,2,3,6,8 | 0,2,3,6,8 | 0,2,3,6,8 | 0,2,3,6,8 | 0,2,3,6,8 | 0,2,3,6,8 | 0,2,3,6,8 | 0,2,3,6,8 | 0,2,3,6,8 | 0,2,3,6,8 | 0,2,3,6,8 |
| 15-3 | 5,10,11 | 5,10,11 | 5,10,11 | 5,10,11 | 5,10,11 | 5,10,11 | 5,10,11 | 5,10,11 | 5,10,11 | 5,10,11 | 5,10,11 |
| 15-4 | 5,6,10,11 | 5,6,10,11 | 5,6,10,11 | 5,6,10,11 | 5,6,10,11 | 5,6,10,11 | 5,6,10,11 | 5,6,10,11 | 5,6,10,11 | 5,6,10,11 | 5,6,10,11 |
| 15-5 | 0,5,6,10,11 | 5,6,10,11 | 5,6,10,11 | 5,6,10,11 | 5,6,10,11 | 5,6,10,11 | 5,6,10,11 | 5,6,10,11 | 5,6,10,11 | 5,6,10,11 | 5,6,10,11 |
| 20-3 | 4,8,17 | 4,8,17 | 4,8,17 | 4,8,17 | 4,8,17 | 4,8,17 | 4,8,17 | 4,8,17 | 4,8,17 | 4,8,17 | 4,8,17 |
| 20-4 | 4,8,13,17 | 4,8,13,17 | 4,8,13,17 | 4,8,13,17 | 4,8,13,17 | 4,8,13,17 | 4,8,13,17 | 4,8,13,17 | 4,8,13,17 | 4,8,13,17 | 4,8,13,17 |
| 20-5 | 1,4,7,8,18 | 1,4,7,8,18 | 1,4,7,8,18 | 1,4,7,8,18 | 1,4,7,8,18 | 1,4,7,8,18 | 1,4,7,8,18 | 1,4,7,8,18 | 1,4,7,8,18 | 1,4,7,8,18 | 1,4,7,8,18 |
| 25-3 | 1,10,19 | 1,10,19 | 1,10,19 | 1,10,19 | 1,10,19 | 1,10,19 | 1,10,19 | 1,10,19 | 1,10,19 | 1,10,19 | 1,10,19 |
| 25-4 | 1,5,10,19 | 1,5,10,19 | 1,5,10,19 | 1,5,10,19 | 1,5,10,19 | 1,5,10,19 | 1,5,10,19 | 1,5,10,19 | 1,5,10,19 | 1,5,10,19 | 1,5,10,19 |
| 25-5 | 0,11,17,20,24 | 0,11,17,20,  24 | 0,11,17,20,  24 | 0,11,17,20,24 | 0,11,17,20,24 | 0,11,17,20,24 | 0,11,17,20,24 | 0,11,17,20,24 | 0,11,17,20,24 | 0,11,17,20,24 | 0,11,17,20,24 |

**Table A3.** Results of the hub solution with constant width and variation of the most probable value.

| **Instance** | **Step Size** | | | | | | | | | | |
| --- | --- | --- | --- | --- | --- | --- | --- | --- | --- | --- | --- |
|  | 0 | 1 | 2 | 3 | 4 | 5 | 6 | 7 | 8 | 9 | 10 |
| 10-3 | 2,3,5 | 3,4,5 | 3,6,8 | 3,6,7 | 3,6,7 | 3,6,7 | 3,6,7 | 3,6,7 | 2,3,8 | 3,6,7 | 3,6,7 |
| 10-4 | 2,3,6,8 | 2,3,6,8 | 2,3,6,8 | 2,3,6,8 | 2,3,6,8 | 2,3,6,8 | 2,3,6,8 | 2,3,6,8 | 2,3,6,7 | 2,3,6,7 | 2,3,6,7 |
| 10-5 | 2,3,6,7,8 | 2,3,6,7,8 | 2,3,6,7,8 | 2,3,6,7,8 | 2,3,6,7,8 | 2,3,6,7,8 | 2,3,6,7,8 | 2,3,6,7,8 | 2,3,6,7,8 | 2,3,6,7,8 | 2,3,6,7,8 |
| 15-3 | 8,10,11 | 8,10,11 | 8,10,11 | 8,10,11 | 8,10,11 | 3,8,11 | 3,8,11 | 3,8,11 | 3,6,11 | 3,6,11 | 3,6,11 |
| 15-4 | 0,8,10,11 | 0,8,10,11 | 6,8,10,11 | 0,3,8,11 | 0,3,8,11 | 0,3,8,11 | 0,3,8,11 | 0,3,8,11 | 3,6,7,11 | 3,6,7,11 | 3,6,7,11 |
| 15-5 | 0,3,8,10,11 | 0,3,8,10,11 | 0,3,5,7,10 | 0,3,8,10,11 | 0,3,6,8,11 | 0,3,6,8,11 | 0,3,6,8,11 | 0,3,6,8,11 | 0,3,6,7,11 | 3,6,8,11,13 | 3,6,8,11,13 |
| 20-3 | 3,17,18 | 3,17,18 | 3,17,18 | 3,17,18 | 3,17,18 | 3,17,18 | 3,17,18 | 3,17,18 | 3,10,17 | 8,10,18 | 8,10,18 |
| 20-4 | 3,4,17,18 | 3,17,18 | 3,17,18 | 3,17,18 | 3,17,18 | 3,17,18 | 3,13,17,18 | 3,13,17,18 | 3,6,8,18 | 3,4,18,19 | 3,13,17,18 |
| 20-5 | 1,3,4,10,17 | 3,4,10,17,18 | 1,3,10,13,17 | 3,4,10,17,18 | 3,4,13,17,18 | 3,10,13,17,18 | 3,4,13,17,18 | 1,3,13,17,18 | 3,6,7,8,18 | 3,4,13,18,19 | 3,8,13,17,18 |
| 25-3 | 1,3,18 | 5,19,24 | 1,10,19 | 1,10,19 | 1,10,19 | 1,10,19 | 1,10,19 | 10,11,19 | 10,11,19 | 3,11,24 | 3,5,18 |
| 25-4 | 1,3,13,18 | 0,10,17,24 | 1,10,11,17 | 3,17,18,23 | 1,10,11,17 | 3,11,17,24 | 1,5,10,19 | 11,10,19,24 | 3,11,13,24 | 3, 7,17,18 | 1.3.6.18 |
| 25-5 | 1,5,10,19,24 | 5,10,19,23,24 | 0,3,17,18,23 | 5,11,19,23,  24 | 5,10,11,19,  24 | 3,10,11,17,  24 | 3,10,11,17,  24 | 10,11,13,19,  24 | 10,11,13,17,19 | 1,3,10,13,19 | 3,7,13,17,18 |

**Table A4.** The result of changing the most probable value of the hub scheme while widening the width.

| **Instance** | **Step Size** | | | | | | | | | | |
| --- | --- | --- | --- | --- | --- | --- | --- | --- | --- | --- | --- |
|  | 0 | 1 | 2 | 3 | 4 | 5 | 6 | 7 | 8 | 9 | 10 |
| 10-3 | 2,3,5 | 3,6,8 | 3,6,7 | 2,3,8 | 2,3,8 | 3,6,7 | 3,6,7 | 3,6,7 | 3,6,7 | 3,6,7 | 3,6,7 |
| 10-4 | 2,3,6,8 | 2,3,6,8 | 2,3,6,7 | 2,3,6,8 | 2,3,6,7 | 2,3,6,7 | 2,3,6,7 | 2,3,6,7 | 2,3,6,7 | 2,3,6,7 | 2,3,6,7 |
| 10-5 | 2,3,6,7,8 | 2,3,6,7,8 | 2,3,6,7,8 | 2,3,6,7,8 | 2,3,6,7,8 | 2,3,6,7,8 | 2,3,6,7,8 | 2,3,6,7,8 | 2,3,6,7,8 | 2,3,6,7,8 | 2,3,6,7,8 |
| 15-3 | 8,10,11 | 3,5,10 | 3,8,11 | 3,6,11 | 3,6,11 | 3,6,11 | 3,6,11 | 3,6,11 | 3,6,11 | 3,6,11 | 3,6,11 |
| 15-4 | 0,8,10,11 | 0,3,5,10 | 3,8,10,11 | 3,8,11,13 | 3,6,7,11 | 3,6,7,11 | 3,6,7,11 | 3,6,7,11 | 3,6,7,11 | 3,6,7,11 | 3,6,7,11 |
| 15-5 | 0,3,8,10,11 | 0,3,8,10,11 | 0,3,8,6,11 | 3,6,8,11,13 | 3,6,8,11,13 | 3,6,8,11,13 | 3,6,8,11,13 | 2,3,6,11,13 | 2,3,6,11,13 | 2,3,6,11,13 | 2,3,6,11,13 |
| 20-3 | 3,17,18 | 3,17,18 | 3,17,18 | 3,17,18 | 3,17,18 | 3,17,18 | 3,10,17 | 3,10,17 | 3,11,17 | 3,11,17 | 3,11,17 |
| 20-4 | 3,4,17,18 | 3,13,17,18 | 3,13,17,18 | 3,13,17,18 | 3,13,17,18 | 3,6,8,18 | 3,13,17,18 | 3,13,17,18 | 3,13,17,18 | 3,8,13,18 | 3,11,13,17 |
| 20-5 | 1,3,4,10,17 | 1,3,10,17,18 | 3,4,13,17,18 | 1,3,13,17,18 | 3,6,11,13,17 | 3,6,8,13,18 | 3,6,13,17,18 | 3,8,13,17,18 | 3,8,13,17,18 | 3,6,8,13,18 | 3,6,11,13,17 |
| 25-3 | 1,3,18 | 1,11,14 | 1,10,19 | 1,10,11 | 10,11,19 | 3,11,24 | 3,11,24 | 3,7,19 | 7,8,17 | 3,8,11 | 3,11,16 |
| 25-4 | 1,3,13,18 | 3,17,18,23 | 10,11,16,24 | 3,11,13,24 | 3,11,16,24 | 3,11,16,24 | 1,3,6,18 | 3,6,8,18 | 1,3,13,18 | 3,6,8,11 | 3,11,16,21 |
| 25-5 | 1,5,10,19,24 | 3,7,17,18,23 | 10,11,13,20,24 | 3,11,13,20,24 | 3,7,13,17,18 | 3,11,13,16,24 | 3,7,13,17,18 | 3,7,13,17,18 | 3,7,13,17,24 | 3,6,8,11,22 | 3,11,13,16,21 |

**Table A5.** The result of changing the most probable value of the hub scheme while narrowing the width.

| **Instance** | **Step Size** | | | | | | | | | | |
| --- | --- | --- | --- | --- | --- | --- | --- | --- | --- | --- | --- |
|  | 0 | 1 | 2 | 3 | 4 | 5 | 6 | 7 | 8 | 9 | 10 |
| 10-3 | 2,3,5 | 3,4,5 | 3,4,5 | 3,6,8 | 3,6,8 | 3,6,8 | 3,6,8 | 3,6,8 | 3,6,8 | 3,6,8 | 3,6,8 |
| 10-4 | 2,3,6,8 | 2,3,6,8 | 2,3,6,8 | 2,3,6,8 | 2,3,6,8 | 2,3,6,8 | 2,3,6,8 | 2,3,6,8 | 2,3,6,8 | 2,3,6,8 | 2,3,6,8 |
| 10-5 | 2,3,6,7,8 | 2,3,6,7,8 | 2,3,6,7,8 | 0,2,3,6,8 | 0,2,3,6,8 | 0,2,3,6,8 | 0,2,3,6,8 | 0,2,3,6,8 | 0,2,3,6,8 | 0,2,3,6,8 | 0,2,3,4,6 |
| 15-3 | 8,10,11 | 8,10,11 | 8,10,11 | 5,10,11 | 5,10,11 | 5,10,11 | 5,10,11 | 5,11,12 | 5,11,12 | 5,11,12 | 5,11,12 |
| 15-4 | 0,8,10,11 | 6,8,10,11 | 6,8,10,11 | 5,6,10,11 | 5,6,10,11 | 5,6,10,11 | 5,6,10,11 | 5,6,10,11 | 5,9,11,12 | 5,9,11,12 | 5,9,11,12 |
| 15-5 | 0,3,8,10,  11 | 0,6,8,10,11 | 0,6,8,10,11 | 0,5,6,10,11 | 0,5,6,10,11 | 0,5,6,10,11 | 0,5,6,10,11 | 0,5,6,10,11 | 0,5,6,10,11 | 0,5,6,10,11 | 5,9,10,11,12 |
| 20-3 | 3,17,18 | 3,17,18 | 3,17,18 | 1,4,8 | 1,4,8 | 1,4,8 | 1,4,8 | 1,4,8 | 1,4,8 | 1,4,8 | 1,4,8 |
| 20-4 | 3,4,17,18 | 3,4,17,18 | 3,4,17,18 | 3,4,17,18 | 3,4,17,18 | 3,4,17,18 | 3,4,17,18 | 1,4,8,17 | 1,4,8,17 | 1,4,8,17 | 1,4,8,17 |
| 20-5 | 1,3,4,10,  s17 | 3,4,10,17,18 | 3,10,12,17,18 | 1,4,7,8,18 | 1,4,7,9,18 | 1,4,7,9,18 | 1,4,7,13,18 | 1,4,7,13,18 | 1,4,7,9,18 | 1,4,7,13,18 | 1,4,7,13,18 |
| 25-3 | 1,3,18 | 3,17,18 | 1,4,24 | 1,10,19 | 1,10,19 | 1,10,19 | 10,19,24 | 10,19,24 | 10,19,24 | 10,19,24 | 10,19,24 |
| 25-4 | 1,3,13,18 | 3,5,17,18 | 5,19,23,24 | 10,19,23,24 | 10,19,23,24 | 1,3,10,19 | 1,3,10,19 | 1,3,10,19 | 10,19,23,24 | 10,19,23,24 | 10,19,23,24 |
| 25-5 | 1,5,10,19,24 | 0,11,17,19,24 | 5,10,19,23,24 | 0,11,17,20,  24 | 0,11,17,20,  24 | 0,11,17,20,  24 | 0,11,17,20,24 | 0,11,17,20,24 | 0,11,17,20,24 | 0,11,17,20,24 | 0,11,17,20,24 |
